# Kinome Analysis of Receptor-Induced Phosphorylation in Human Natural Killer Cells

**DOI:** 10.1371/journal.pone.0029672

**Published:** 2012-01-04

**Authors:** Sebastian König, Manfred Nimtz, Maxi Scheiter, Hans-Gustaf Ljunggren, Yenan T. Bryceson, Lothar Jänsch

**Affiliations:** 1 Department of Molecular Structural Biology, Helmholtz-Zentrum für Infektionsforschung, Braunschweig, Germany; 2 Center for Infectious Medicine, Department of Medicine, Karolinska Institutet, Karolinska University Hospital Huddinge, Stockholm, Sweden; Deutsches Krebsforschungszentrum, Germany

## Abstract

**Background:**

Natural killer (NK) cells contribute to the defense against infected and transformed cells through the engagement of multiple germline-encoded activation receptors. Stimulation of the Fc receptor CD16 alone is sufficient for NK cell activation, whereas other receptors, such as 2B4 (CD244) and DNAM-1 (CD226), act synergistically. After receptor engagement, protein kinases play a major role in signaling networks controlling NK cell effector functions. However, it has not been characterized systematically which of all kinases encoded by the human genome (kinome) are involved in NK cell activation.

**Results:**

A kinase-selective phosphoproteome approach enabled the determination of 188 kinases expressed in human NK cells. Crosslinking of CD16 as well as 2B4 and DNAM-1 revealed a total of 313 distinct kinase phosphorylation sites on 109 different kinases. Phosphorylation sites on 21 kinases were similarly regulated after engagement of either CD16 or co-engagement of 2B4 and DNAM-1. Among those, increased phosphorylation of FYN, KCC2G (CAMK2), FES, and AAK1, as well as the reduced phosphorylation of MARK2, were reproducibly observed both after engagement of CD16 and co-engagement of 2B4 and DNAM-1. Notably, only one phosphorylation on PAK4 was differentally regulated.

**Conclusions:**

The present study has identified a significant portion of the NK cell kinome and defined novel phosphorylation sites in primary lymphocytes. Regulated phosphorylations observed in the early phase of NK cell activation imply these kinases are involved in NK cell signaling. Taken together, this study suggests a largely shared signaling pathway downstream of distinct activation receptors and constitutes a valuable resource for further elucidating the regulation of NK cell effector responses.

## Introduction

Natural killer (NK) cells are lymphocytes belonging to the innate immune system. They can eliminate infected or transformed cells through direct killing of target cells [1]. Moreover, NK cells also influence cells of the adaptive immune system through release of chemokines and cytokines, as well as by contact-dependent killing of activated immune cells [2], [3]. The activation and effector functions of NK cells are controlled by signals from a multiplicity of germline-encoded activating and inhibitory receptors [4], [5]. Downstream of receptor engagement, the underlying signaling networks in NK cells are controlled by protein kinases [6], [7], [8], which constitute the largest family of enzymes in the human genome [9]. More than 500 different protein kinases coordinate steps in virtually all intra-cellular signaling pathways. Generally, protein kinases are expressed at relatively low levels and require targeted approaches for their direct characterization [10]. Dynamic post-translational modifications, including phosphorylations, regulate the enzymatic activity, localization, and substrate binding competence of kinases.

In terms of signaling by NK cell receptors, structurally distinct inhibitory receptors all contain immunoreceptor tyrosine-based inhibition motifs (ITIMs). The signaling by such motifs has been extensively studied and is mediated by activation of tyrosine phosphatases as well as the tyrosine kinase c-Abl [11], [12]. In contrast, activating NK cells receptors have highly divergent cytoplasmic signaling domains, and signaling pathways orchestrated by many activating NK cell receptors are not well defined. The low affinity Fc receptor CD16 (FcγRIIIA) is the prototypical NK cell activating receptor. Engagement of CD16 induces SRC tyrosine kinase-dependent phosphorylation of immunoreceptor tyrosine-based activation motifs (ITAM) on the adaptor chains CD3ζ and FcεRγ, which in turn recruit and activate SYK and ZAP-70 tyrosine kinases for downstream signaling [13]. Engagement of CD16 on NK cells is sufficient to induce NK cell degranulation for antibody-dependent cellular cytotoxicity (ADCC) [14]. Since several features of proximal CD16 signaling are shared with that of the T cell receptor (TCR), downstream events are thought to resemble those characterized in T cells. However, signaling induced by different ITAM-coupled receptors has not been rigorously compared. For natural cytotoxicity, engagement of individual receptors normally does not suffice, whereas co-engagement of specific pairwise combinations of receptors can induce synergistic intracellular Ca^2+^ mobilization as well as degranulation and cytokine production [15], [16]. Thus, receptors such as 2B4 (CD244) and DNAM-1 (CD226) have been termed “co-activating receptors” [15]. 2B4 binds CD48 [17], a ligand ubiquitously expressed on hematopoeitic cells. 2B4 contains cytoplasmic immunoreceptor tyrosine-based switch motifs (ITSMs). These motifs are phosphorylated and can recruit either phosphatases for negative regulation of NK cell functions, or, via the adaptor SAP, the SRC kinase FYN for the activation of NK cells [18], [19], [20]. In human NK cells, 2B4 is activating unless cells are deficient in SAP, which is the case in the primary immunodeficiency X-linked lymphoproliferative type 1 caused by mutations in *SH2D1A* whereupon engagement of 2B4 results in inhibitory signals [18], [21]. DNAM-1 binds the widely expressed nectins CD112 and CD155 [22]. The cytoplasmic domain of DNAM-1 is phosphorylated by protein kinase C (PKC) [23]. Further downstream, signaling by DNAM-1 in NK cells is not well understood but involves also serine and threonine phosphorylations rarely characterized in NK biology [24], [25]. In terms of co-activating receptor synergy, a recent report suggests that, rather than complementary signaling between different receptors, the basis for co-activation is dependent on a summation of signals from each receptor for VAV1 activation to overcome an inhibitory threshold set by c-Cbl [26].

In conclusion, further studies focusing at signaling downstream of co-activating receptors and the prototypical NK cell activation receptor CD16 are needed to identify common nodes as well as pathway specificities of receptors with distinct cytoplasmic domains, helping to define the core-signal network regulating NK cell activation.

Mass spectrometry (MS) can provide insights into protein phosphorylation events that coordinate signaling networks. In particular, Fourier transform mass spectrometry (FTMS) routinely achieves a very high data accuracy, facilitating unambiguous protein sequencing and identification of amino acid site-specific post-translational modifications [27]. Phosphoproteome studies have already been performed on T cell lines, shedding light on TCR signaling networks [28], [29], [30]. However, due to the requirement for large cell amounts, only a few MS-based studies have used primary lymphocytes [31]. Gaining access to signaling components, such as protein kinases, is more methodologically demanding and requires, in addition to significant amounts of starting cell material, efficient pre-enrichment of target proteins. Here, we use a kinase-selective phosphoproteome strategy to probe receptor-induced kinase phosphorylation in primary human NK cells. The results characterize the NK cell kinome including the first unbiased knowledge about its phosphorylation. Quantitative MS data reveal novel kinase phosphorylation sites that are modulated following CD16 activation or co-engagement of 2B4 and DNAM-1. Notably, the regulatory patterns were remarkably similar following activation through such distinct receptors, suggesting a largely shared signal network. To our knowledge, this study is the first proteome approach that specifically determines receptor-dependent kinase phosphorylation in primary lymphocytes.

## Results

### Expansion and characterization of primary NK cells

**Figure 1:**
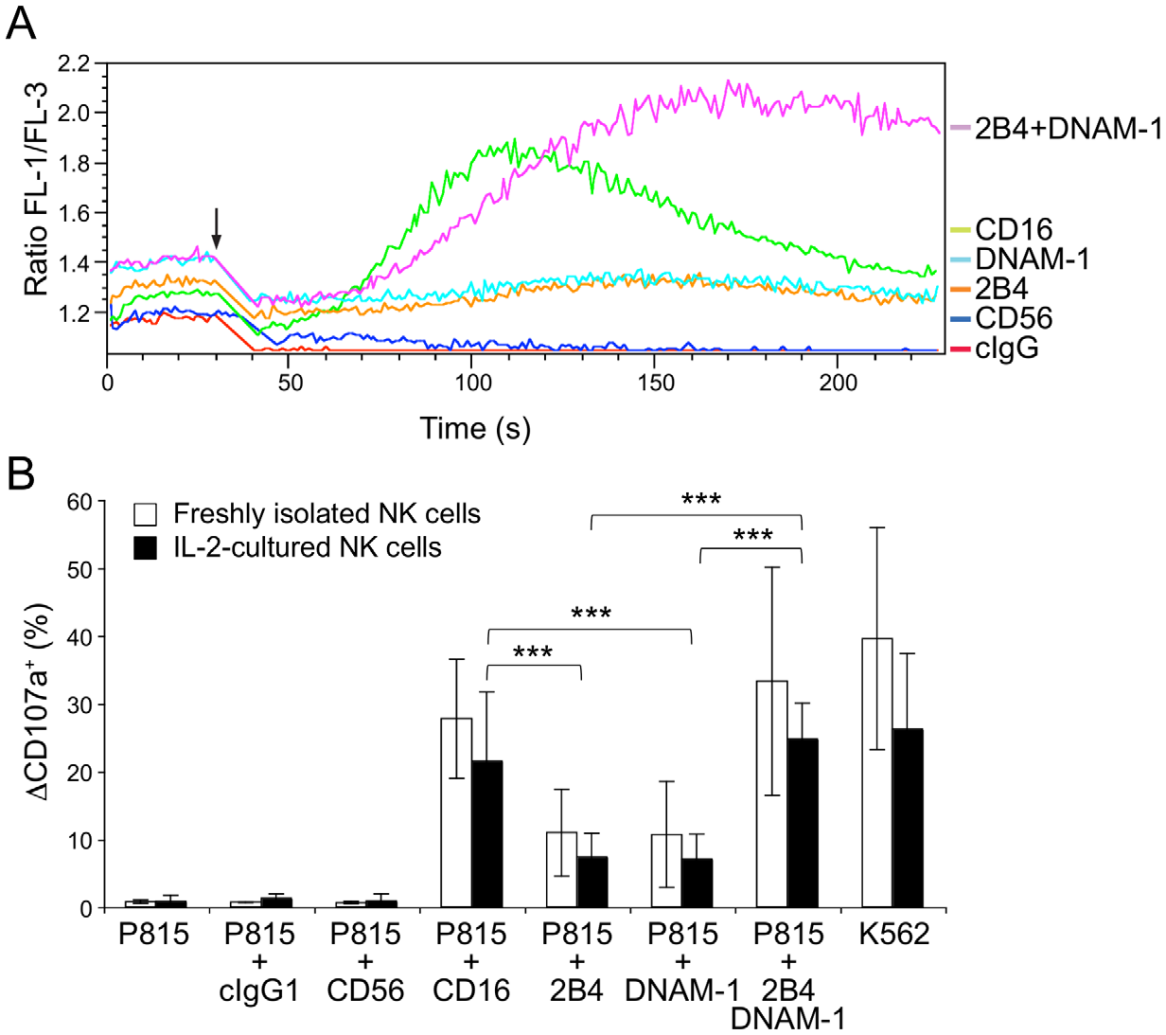
Functional characterization of IL-2–cultured primary NK cells. (**A**) Intracellular Ca^2+^-mobilization induced by activating NK cell receptors. The following monoclonal antibodies (mAbs) were used for NK cell stimulation: negative controls, IgG isotype (cIgG, red) and anti-CD56 (dark blue); receptor stimulation, anti-2B4 (orange), anti-DNAM-1 (light blue), anti-CD16 (green) and anti-2B4 and anti-DNAM-1 (purple). Polyclonal IL-2–cultured NK cells were pre-incubated with the indicated mAbs on ice and loaded with Fluo-4 and Fura Red dyes. Ca^2+^-mobilization was assessed by flow cytometry. After 30 sec, secondary F(ab′)2 goat anti-mouse IgG crosslinking antibody was added (black arrow). FL-1/FL-3 ratios are plotted as a function of time. (**B**) Comparison of degranulation by freshly isolated (open bars) and IL-2–cultured (black bars) NK cells. NK cells were incubated with the following target cells: murine P815 cells (negative control), P815 cells coated with mAbs to the indicated receptors (Fc receptor-positive for redirected ADCC; IgG isotype and anti-CD56 mAb coated P815 cells were used as negative controls) or with NK cell-susceptible human K562 cells (positive control). Subsequently, NK cells were stained with fluorochrome-conjugated anti-CD56 and anti-CD107a mAbs and analyzed by flow cytometry. The percentage of CD107a-positive NK cells is shown as mean ± standard deviation (SD) of at least eight independent experiments. Differences between two groups were examined using the Student's t-test (***, p<0.001). Correlation between degranulation by freshly isolated (open bars) and IL-2–cultured (black bars) NK cells were determined according to Pearson (r = 0.95).

To obtain sufficient material for quantitative proteome studies, NK cells were isolated from the peripheral blood of healthy human donors and polyclonally expanded with irradiated, allogeneic feeder cells and recombinant IL-2 (Figure S1). To ensure that no gross perturbations in NK cell function occurred upon NK cell expansion, the readouts of early signaling events in IL-2–cultured NK cells were compared to those of freshly isolated NK cells (Figure 1). Initially, NK cells were labeled with Ca^2+^-sensitive fluorescent dyes and incubated either with IgG isotype control, anti-CD56 (both served as negative controls), anti-CD16, anti-2B4, or anti-DNAM-1, as well as co-incubated with anti-2B4 and anti-DNAM-1 monoclonal antibodies (mAbs) on ice. Thereafter, cells were pre-warmed for 5 minutes at 37°C and analyzed on a flow cytometer. The strength and dynamics of intracellular Ca^2+^-mobilization were assessed in NK cells following crosslinking with a secondary goat anti-mouse Fc antibody (Figure 1A). Whereas isotype control mAbs or crosslinking of CD56 did not elicit any Ca^2+^-mobilization in IL-2–cultured NK cells, crosslinking of CD16 induced a robust mobilization of intracellular Ca^2+^ similar, albeit diminished in magnitude, to that observed in freshly isolated NK cells [15]. Crosslinking of either 2B4 or DNAM-1 did not induce Ca^2+^-mobilization in IL-2–cultured NK cells, as previously observed for freshly isolated NK cells [15]. However, co-crosslinking of 2B4 and DNAM-1 synergized to produce intracellular Ca^2+^-mobilization in IL-2–cultured NK cells, again as previously described in freshly isolated NK cells [15]. Next, we compared receptor-induced degranulation in IL-2–cultured and freshly isolated NK cells (Figure 1B). NK cell-susceptible human K562 cells and murine P815 cells (Fc receptor-positive for redirected ADCC) were used as target cells. NK cells were incubated with target cells and mAbs, as indicated, for one hour followed by staining and analysis by flow cytometry. Degranulation was quantified in terms of induced CD107a surface expression. IL-2–cultured or freshly isolated NK cells did not degranulate in response to P815 cells or P815 cells co-incubated with isotype control or anti-CD56 mAb. However, engagement of CD16 induced degranulation of both IL-2–cultured and freshly isolated NK cells. Of note, degranulation was somewhat stronger in freshly isolated NK cells, consistent with stronger Ca^2+^-mobilization in freshly isolated NK cells. Co-engagement of 2B4 and DNAM-1 resulted in synergistic degranulation by both IL-2–cultured and freshly isolated NK cells, and degranulation induced by incubation with K562 cells was also similar between IL-2–cultured and freshly isolated NK cells. Moreover, comparison of receptor expression by IL-2–cultured and freshly isolated NK cells have previously revealed similar expression levels [15]. Taken together, the IL-2–cultured NK cells retained sufficient functional and phenotypic attributes of freshly isolated NK cells and were thus well suited for studies of signal transduction events by quantitative proteomics.

### Kinase-selective phosphoproteome analyses of activated NK cells

**Figure 2:**
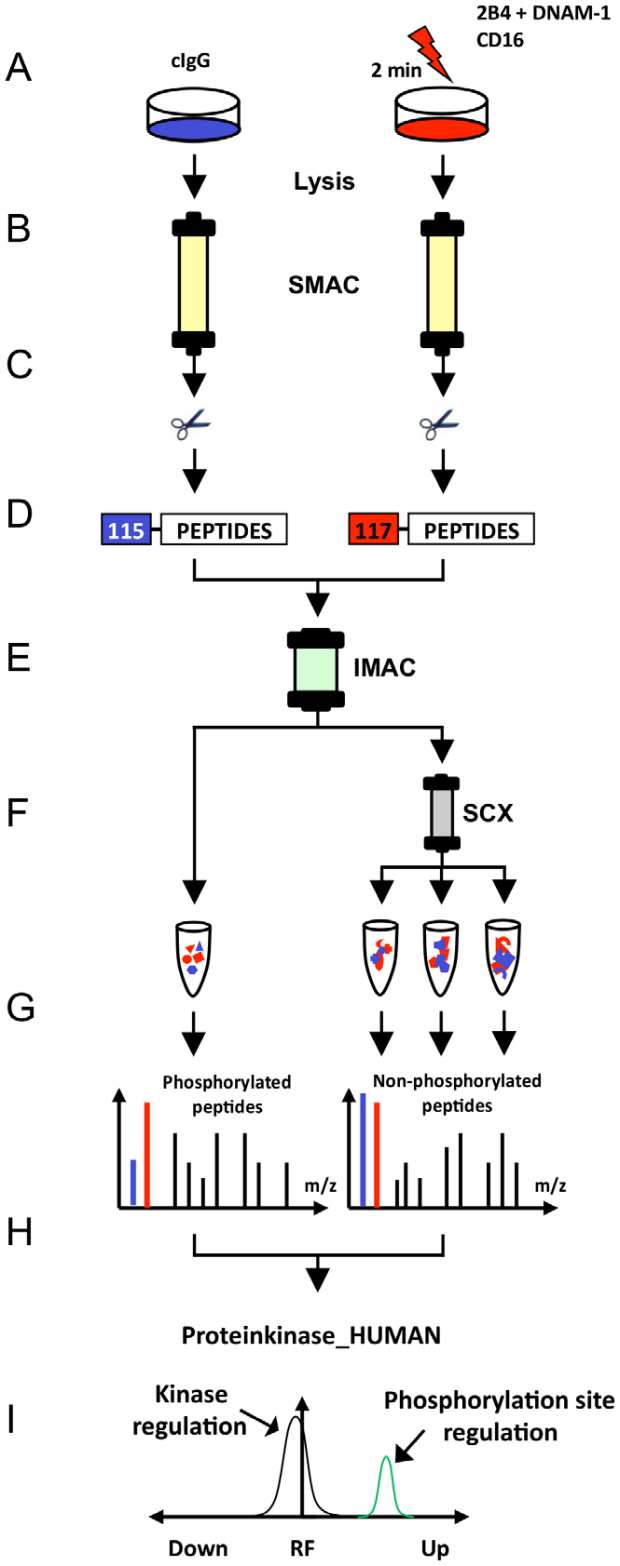
Proteome workflow for the detection of receptor-induced kinase phosphorylation in NK cells. (**A**) Human, polyclonal, IL-2–cultured NK cells were stimulated each with the indicated receptor-specific monoclonal antibodies (mAbs) for 2 minutes (right side) and in all cases were comparatively analyzed with non-stimulated cells treated with control IgG isotype mAbs (cIgG) (left side). (**B**) Protein kinases were purified from total lysates using VI16743/Purvalanol-B-based small molecule affinity chromatography (SMAC). (**C**) Kinase elution and digestion into tryptic peptides comprising all non-modified and phosphorylated peptides. (**D**) Differential peptide labeling with iTRAQ reagents for MS-based quantification of phosphorylation site regulation and combination of samples. (**E**) Enrichment of phosphorylated peptides (phosphopeptides) by immobilized metal affinity chromatography (IMAC). (**F**) Further fractionation of non-phosphorylated peptide samples by strong cation exchange chromatography (SCX). (**G**) Peptide sequence analysis (LC-MS/MS) of all peptide fractions. (**H**) Peptide, kinase and phosphorylation site identification by Mascot database search and manual MS data inspection. (**I**) Statistical evaluation of significantly regulated phosphorylation sites on protein kinases by the MS-specific noise model iTRAQassist [38].

Kinase phosphorylation in receptor-activated and IgG isotype control-treated NK cells were comparatively analyzed by the quantitative proteome workflow depicted in Figure 2. In total, three independent phosphokinome analyses were performed (two included CD16 engagement, all three included 2B4 and DNAM-1 co-engagement; Table S1). IL-2–cultured NK cells were incubated with IgG isotype control or indicated receptor-specific mAbs on ice, pre-warmed to 37°C and crosslinked for 2 minutes (Figure 2A). Notably, at this time-point the strength of intracellular Ca^2+^-mobilization induced by engagement of CD16 or co-engagement of 2B4 and DNAM-1 was similar in magnitude (Figure 1A). After stimulation, cell lysates were generated and protein kinases were enriched as described in the following.

**Figure 3:**
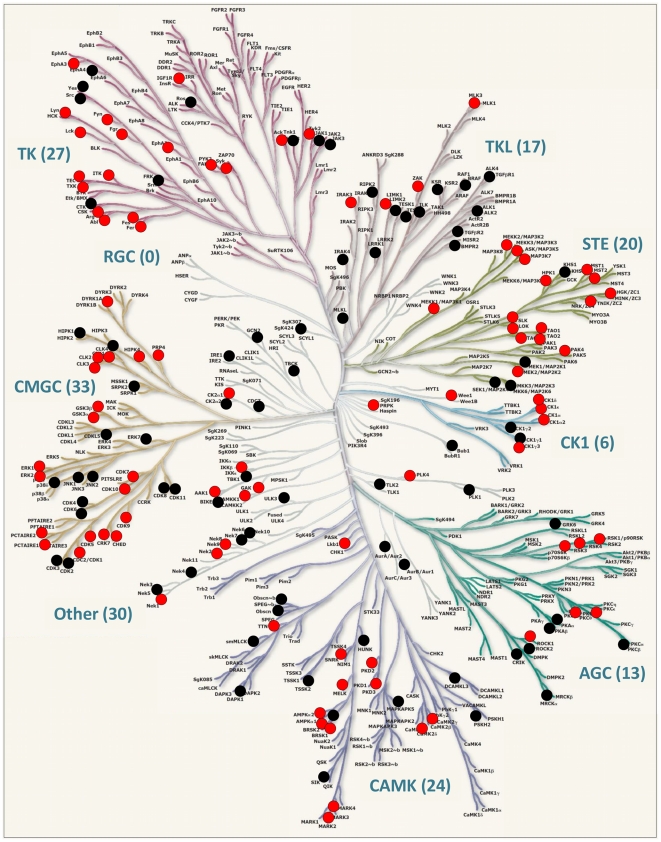
Protein kinases (kinome) expressed in human NK cells. The figure shows the human kinome dendrogram [9]. Kinase-selective proteomics revealed the expression of 170 protein kinases (black and red circles), 5 atypical kinases (not depicted) and 13 non-protein kinases (NPKs, not depicted). Phosphorylation site-specific information was obtained for 95 kinases (red circles), whereas no such information was obtained for the remaining kinases (black circles). Abbreviations: AGC, PKA/PKG/PKC-family kinases; CAMK, calcium/calmodulin-dependent kinases; CK1, casein kinases; CMGC, CDK/MAPK/GSK3/CLK-family kinases; RCG, receptor guanylate cyclases; STE, sterile homologue kinases; TK, tyrosine kinases; TKL, tyrosine kinase-like kinases; atypical protein kinases; Other, belonging to non of the mentioned groups. Human kinome provided courtesy of Cell Signaling Technology, Inc. www.cellsignal.com.

Sample complexity and high abundant proteins usually hamper the systematic characterization of protein kinases by MS. Thus, pre-enrichment of protein kinases from total cell lysates is required. Immobilized ATP-competitive small kinase inhibitors with a broad inhibitory spectrum can facilitate this step prior to proteomic characterization [10], [32], [33], [34]. Here, we used a serial combination of the kinase inhibitors VI16743 and Purvalanol-B that target a significant portion of the human kinome [10], [32], [35], [36]. Lysates generated from IL-2–cultured NK cells stimulated by indicated receptors were subjected to small molecule affinity chromatography (SMAC) for enrichment of protein kinases (Figure 2B). Proteome analyses of the SMAC eluate fractions revealed that approximately 30% of all identified proteins could be classified as kinases according to the Swiss-Prot protein knowledgebase (Figure S2A). Thus, kinases were the most abundant proteins in the eluted fractions and were generally characterized by a significant higher number of MS spectra and corresponding peptide sequences than non-kinases (Figure S2B). These results underline the efficient enrichment of protein kinases from total cell lysates using immobilized kinase inhibitors VI16743 and Purvalanol-B. In total, the expression of 175 protein kinases and 13 non-protein kinases (NPKs, kinases that have low molecular weight phosphate acceptors as substrates) was unambiguously determined in human NK cells (Table S2). According to a large-scale analysis of the human transcriptome suggesting the expression of around 300 distinct kinases in human cells [37], this chemical proteome approach characterized approximately 60% of the kinases expressed in human NK cells. Moreover, an alignment with the human kinome dendrogram [9] showed that kinases from almost all branches of the human kinome were detected in IL-2–cultured NK cells (Figure 3).

**Figure 4:**
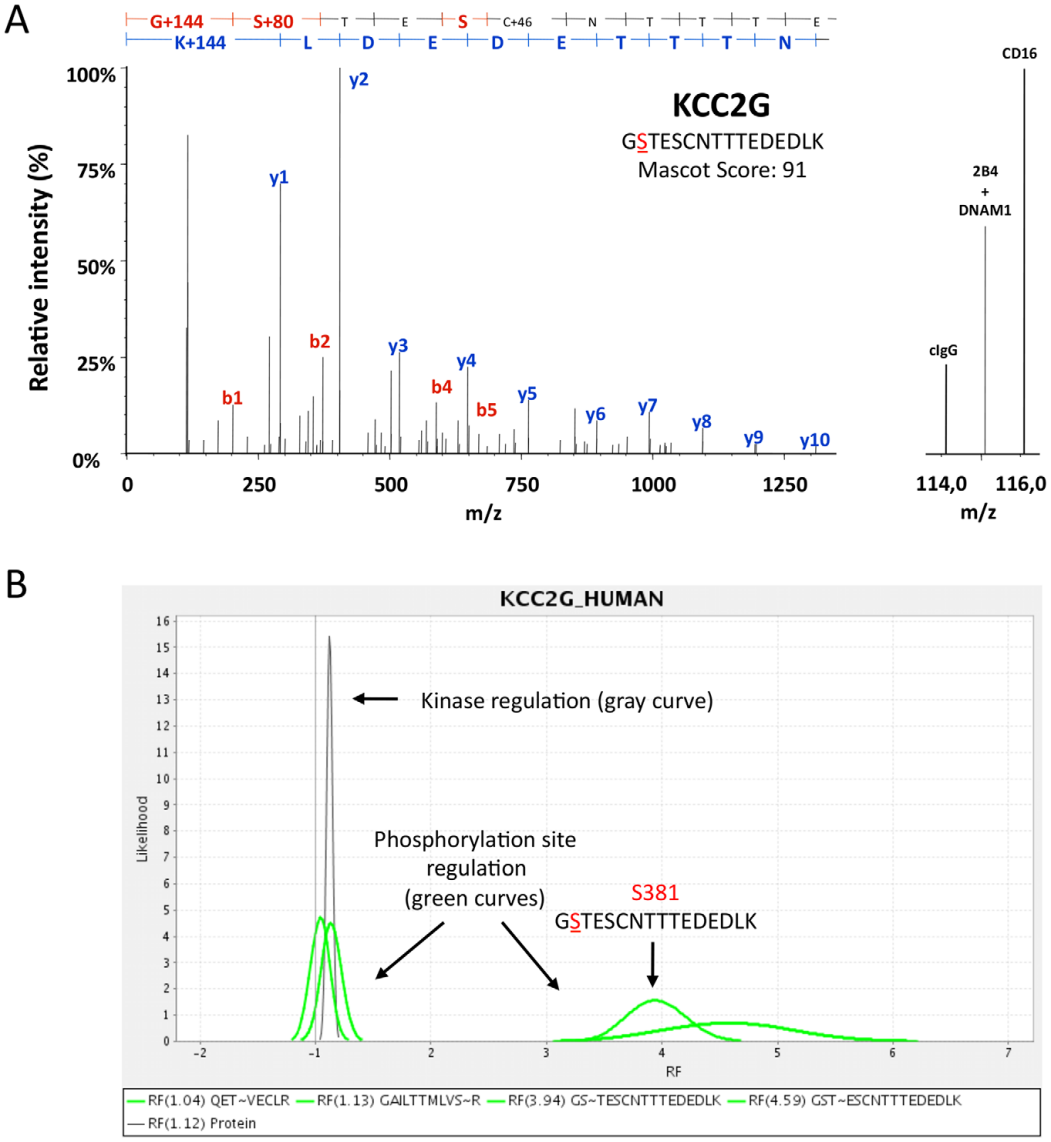
Quantification of kinase phosphorylation induced by activating NK cell receptors. ITRAQ-based quantification and statistical analysis of kinase phosphorylation by iTRAQassist is exemplified for CD16- or 2B4 and DNAM-1-mediated phosphorylation of calcium/calmodulin-dependent protein kinase II gamma (KCC2G). (**A**) MS/MS fragmentation spectrum of a tryptic phosphopeptide derived from KCC2G. Peptide sequencing, protein identification and phosphorylation site annotation are based on fragment ions of the b- (red) and y- (blue) series. The sequence of the phosphopeptide (GSTESCNTTTEDEDLK) is shown in the upper part of the diagram. On the right, a magnification of the low molecular mass range is shown. The intensity of the iTRAQ reporter 114 correlates with the abundance of the respective phosphopeptide in IgG isotype control-treated (cIgG) NK cells, whereas the intensities of the 115 and 116 peaks represent 2B4 and DNAM-1 co-stimulated or CD16-stimulated cells. The MS/MS spectrum is derived from experiment II (see Table S1). (**B**) Statistical evaluation of CD16-induced KCC2G phosphorylation by iTRAQ assist. Statistical analyses of phosphopeptide regulation were performed by iTRAQassist as described previously [38]. Most and less likely peptide regulations (x-axis: regulation factors, RF) were calculated and depicted as likelihood curves (y-axis) for individual phosphopeptides (green curves). The regulation of non-phosphorylated peptides assigned to KCC2G were calculated cumulatively and the resulting protein curve characterizes the general kinase abundance (expression) under the condition of stimulation (gray curve, “kinase regulation”). KCC2G was equally expressed in IgG isotype control-treated and CD16-stimulated NK cells. Only phosphorylation sites having regulation curves clearly separated from the protein curve were considered as regulated. CD16 stimulation (2 min) led to KCC2G phosphorylation on S381 and T382, but not on T287 (QETVECLR) or S311 (GAILTTMLVSR).

Next, all proteins of kinase-enriched SMAC-fractions were digested into peptides and differentially labeled with isobaric iTRAQ reagents (Figure 2C, D) required for peptide quantification by MS (for details, see Materials and methods). Differentially iTRAQ-labeled samples obtained from the respective stimulations were combined and phosphopeptides were purified from kinome-derived peptide mixtures by immobilized metal affinity chromatography (IMAC) to ascertain their optimal characterization by MS (Figure 2E). Phosphopeptide-depleted fractions comprising IMAC flow-through and wash fractions were further sub-fractionated by strong cation exchange (SCX) chromatography (Figure 2F) to reduce ion suppression effects in MS and to improve kinase sequence coverage. LC-MS/MS analyses (Figure 2G) were performed with all fractions and provided fragmentation data on both non-modified peptide sequences as well as those carrying phosphorylations on serine, threonine, or tyrosine residues (see Table S1 – Performed phosphokinome experiments). Since iTRAQ reporter ions are cleaved from each peptide under the condition of peptide sequencing simultaneously, each individual tandem MS fragment ion spectrum also provides quantitative information (Figure 2H, I). Figure 4 shows a representative MS/MS fragmentation spectra that depicts the phosphorylation of KCC2G on S381 induced by engagement of CD16 or co-engagement of 2B4 and DNAM-1: The detected molecular masses of b- (red) and y- (blue) fragment ions identify the phosphopeptide GS∼TESCNTTTEDEDLK, which can be exclusively assigned to the kinase KCC2G (Figure 4A). iTRAQ-reporter ions appeared in the low molecular mass region of this MS/MS spectrum indicating the relative abundance of this phosphorylated peptide following the activation of NK cells by engagement of CD16 (iTRAQ reporter 116) or co-engagement of 2B4 and DNAM-1 (iTRAQ reporter 115) in comparison to the isotype control stimulation (iTRAQ reporter 114). The intensities of the iTRAQ reporter masses 115 and 116 Dalton (Da) confirmed that phosphorylation on S381 was induced by CD16 engagement as well as 2B4 and DNAM-1 co-engagement. An example of the statistical evaluation of receptor-induced kinase phosphorylations by the MS-specific noise model iTRAQassist [38] is shown for CD16-induced phosphorylation of KCC2G (Figure 4B). Curves indicating most and less likely regulations, which must be considered based on the experimental results. Distinct and non-overlapping curves basically indicated significant regulations in stimulated NK cells. These so-called likelihood curves were calculated for each individual phosphopeptide (green curve) and comparatively inspected with a protein regulation curve summarizing quantitative data of all non-phosphorylated KCC2G peptides (gray curve). Phosphopeptides (phosphorylation sites) were only accepted as differentially regulated upon receptor engagement if their likelihood curves were clearly separated from the protein regulation curve (Figure S3).

In this way, all 313 phosphorylation sites identified at 95 distinct kinases (Table S3) were inspected quantitatively. Of those, 102 were detected in all experiments (Figure S4). In total, 22 tyrosine, 62 threonine, and 229 serine phosphorylation events were observed. Thereof, 47 phosphorylations could not be assigned unambiguously to an individual threonine/serine residue as indicated by alternative phosphopeptide sequence annotations. As expected, the majority of peptides, in particular the non-modified peptide sequences, were not regulated significantly following receptor activation. Cluster analyses of all regulation data obtained from non-phosphorylated peptides showed a normal distribution, whereas regulation factors of phosphorylated peptides were distributed non-normally and exhibited a clear shift toward positive regulation values (Figure S5).

**Figure 5:**
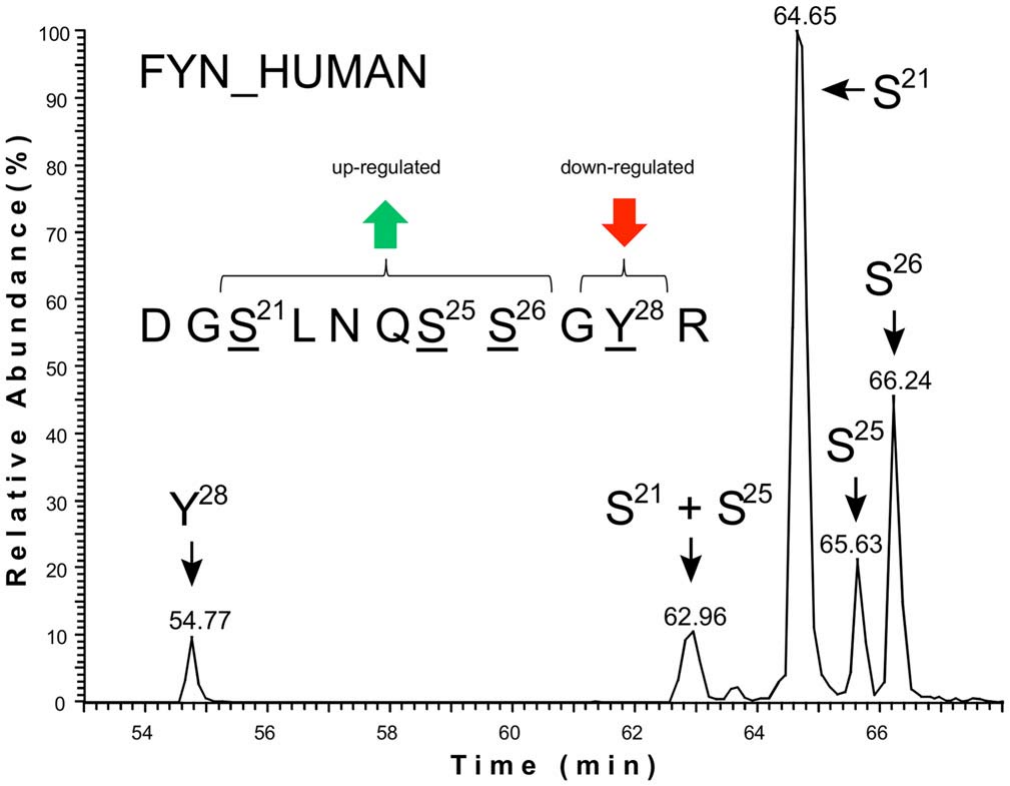
Identification of differentially phosphorylated FYN subsets in NK cells. Nano ultra performance liquid chromatography (nano-UPLC) dissects distinct phosphorylation events at the same peptide DGSLNQSSGYR derived from the protein kinase FYN. MS analyses determined phosphorylations at the amino acid residues S21, S25, S26 and Y28. Successive elution of distinct phosphopeptides based on the same amino acid sequence facilitated the precise quantification of each phosphorylation event at FYN by MS (Figure S6). All phosphorylated serines showed a significantly induced phosphorylation following receptor engagements. However, the relative signal intensities of the distinct phosphopeptide populations indicate a predominant abundance of FYN phosphorylated on S21.

Particular attention was paid to the validation of significantly regulated phosphorylation sites determined by iTRAQassist [38], which also required a distinct separation of individual phosphopeptide populations. Nano ultra performance liquid chromatography (nano-UPLC) could separate peptides based on their sequence and number of modifications, but as is noteworthy, also according to the specific site of phosphorylation. Thus, subpopulations of isobaric phosphopeptides, such as the peptide DGSLNQSSGYR originating from FYN, could be quantified individually. Differentially phosphorylated peptide fractions representing the DGSLNQSSGYR region of FYN were clearly resolved by our chromatography and thus could be characterized separately by MS (Figure 5). This allowed the annotation and quantification of individual phosphorylation sites, even if modifications were located in close proximity. In the case of FYN, the discrimination of two N-terminal regions (S21-S25-S26 and Y28), inversely regulated by CD16 stimulation, was achieved (Figure 5 and Figure S6). Similarly, FYN was phosphorylated on the N-terminal amino acids S21, S25 and S26 following co-engagement of 2B4 and DNAM-1 (Figure S6). However, FYN exclusively phosphorylated at S21 constituted the most prominent part of the responding populations (Figure 5), whereas relatively small amounts of FYN were phosphorylated at S25, S26 and in particular Y28. Taken together, iTRAQassist-based statistical evaluation of quantitative MS data complemented by manual inspection of phosphopeptide elution profiles and fragmentation spectra assured the significance of NK cell receptor-induced kinase modifications in this study.

### Kinase phosphorylations induced by CD16 and co-engagement of 2B4 and DNAM-1

**Figure 6:**
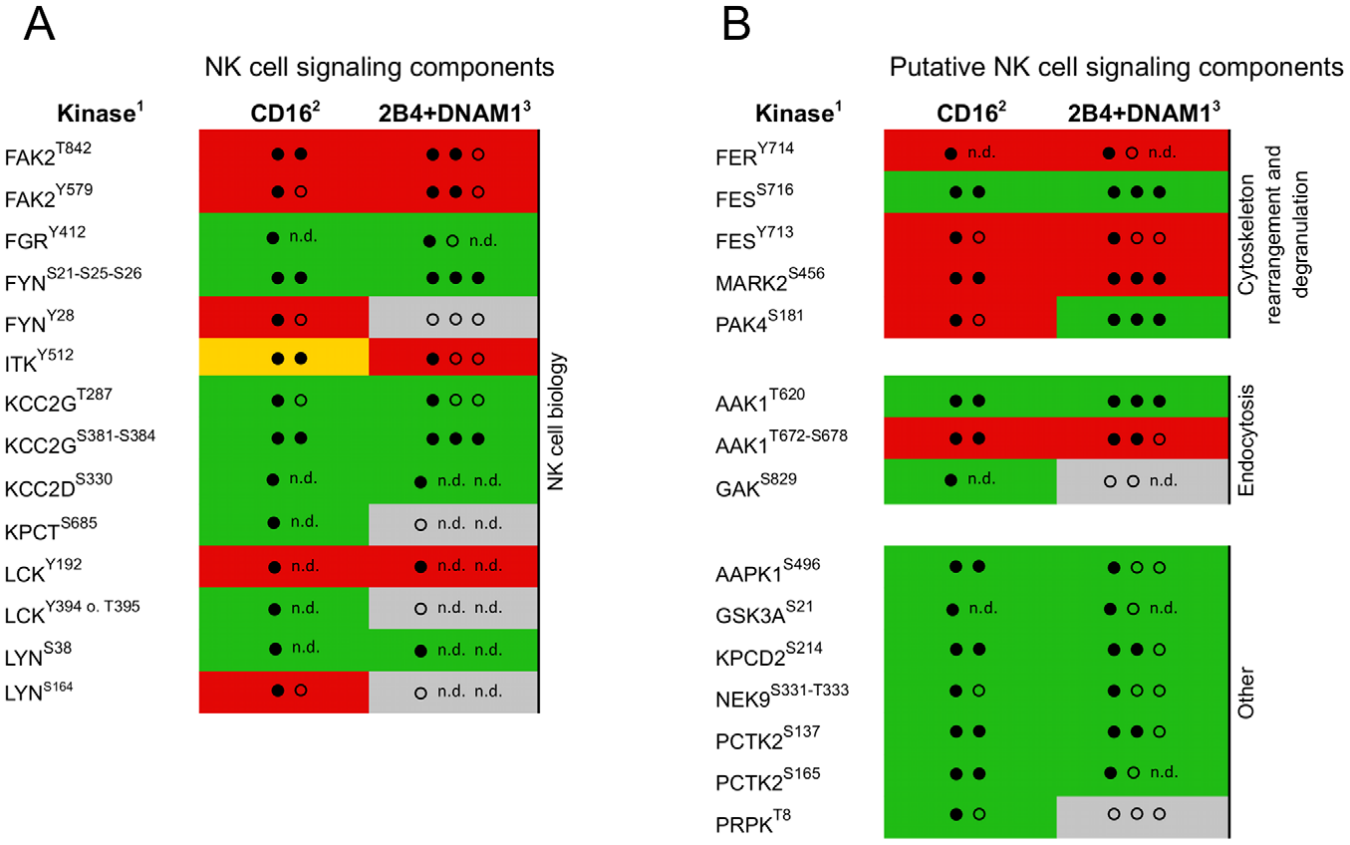
Kinase phosphorylation induced by engagement of CD16 or co-engagement of 2B4 and DNAM-1. CD16- (two proteome experiments) or 2B4 and DNAM-1 co-activated (three proteome experiments) NK cells from two healthy human donors were analyzed in this study. (**A**) Kinases, which have previously been associated with NK cell signaling and/or function. (**B**) Putative novel CD16- and/or 2B4 and DNAM-1-dependent signaling components. ^1)^UniProt name of kinases and phosphorylation site annotation; regulation of phosphorylation site following ^2)^CD16 engagement or ^3)^2B4 and DNAM-1 co-activation is indicated as follows: green, up-regulated; red, down-regulated; gray, no statistical significant phosphorylation site regulation was detected; Number of (filled) circles indicates how often the significant regulation of a respective phosphorylation site was detected according to iTRAQassist (no overlap of protein and respective phosphopeptide regulation curves). Phosphorylation sites regulated with the same tendency, but not matching the criteria of statistical significance are marked as open circles. Proteome experiments that failed to detect a respective phosphorylation site are indicated by n.d. (not detected).

Kinase-mediated signaling downstream of NK cell activation receptors had not been systematically investigated. Therefore, we used NK cells from two human donors and comparatively studied kinase phosphorylation induced by CD16 and co-engagement of 2B4 and DNAM-1. These experiments revealed 21 protein kinases with altered phosphorylation sites after 2 minutes of CD16 engagement (Table 1 and Figure S3). Several phosphorylation sites could not observed by MS under all conditions, which might either reflect the limited sensitivity of this study or indicate donor variations. However, the increased phosphorylation of FYN, KCC2G, FES, and AAK1, as well as the reduced phosphorylation of MARK2, were reproducibly observed in all experiments. 2B4 and DNAM-1 co-engagement generally led to a slightly weaker phosphorylation of these phosphorylation sites, and according to the statistical noise model iTRAQassist, some regulations did not achieve a significant regulation score. However, with few exceptions, phosphorylation induced by 2B4 and DNAM-1 co-engagement was in accordance with that induced by CD16 engagement (Figure 6).

**Table 1:** Kinase phosphorylation induced by CD16 (2 min).

Kinase^1^	Phosphorylation-site function^2^ and regulation^3^		Function^4^	References^5^
NK cell function^$^				
FAK2^T842^, FAK2^Y579^	N/K, N/K	down, down	Natural cytotoxicity, integrin signaling, IL15-mediated NK cell priming	[69] [70]
FGR^Y412^	(A)	up	IFNγ production	[71]
FYN^S21-S25-S26^, FYN^Y28^	A/N/K/*, (A)	up, down	2B4 signaling, LFA-1-dependent DNAM-1 phosphorylation	[19] [45] [46] [25]
ITK^Y512^	A	donor-dependent	Fc receptor and NKG2D signaling	[72]
KCC2G^T287^, KCC2G^S381–S384^	(A), N/K	up, up	LFA-1-dependent NK cell activation by DCs	[50]
KCC2D^S330^	N/K	up	LFA-1-dependent NK cell activation by DCs	[50]
KPCT^S685^	N/K	up	NK cell activation, CD16 and 2B4 signaling	[73] [74] [75] [76] [77] [78] [79]
LCK^Y192^, LCK^Y394 or T395^	I, A/*	down, up	NK cell activation, CD16 signaling	[80] [81] [82] [83]
LYN^S38^, LYN^S164^	*, *	up, down	CD16 signaling	[81]
Cytoskeleton rearrangement and degranulation^§^				
FER^Y714^	A	down	Cytoskeleton dynamic, FcεRI signaling	[62] [63]
FES^S716^, FES^Y713^	N/K, A	up, down	Cytoskeleton dynamic, FcεRI signaling, vesicle trafficking and degranulation	[62] [63] [84] [64]
MARK2^S456^	N/K	down	Mikrotubuli reorganization, granula exocytosis	[66] [85]
PAK4^S181^	N/K	down	Actin cytoskeleton dynamic, adhesion	[43] [44]
Clathrin-mediated endocytosis^§^				
AAK1^T620^, AAK1^T672-S678^	N/K, N/K	up, down	Clathrin-mediated endocytosis	[86] [87] [88]
GAK^S829^	N/K	up	Clathrin-mediated endocytosis	[86] [87] [88]
Other^§^				
AAPK1^S496^	(A)	up	ATP-dependent biosynthesis	[89] [90]
GSK3A^S21^	I	up	PI3K/Akt-, NFAT signaling	[91] [92]
KPCD2^S214^	N/K	up	DAG/phorbol ester, NFAT signaling	[93] [94]
NEK9^S331-T333^	N/K	up	Cell cycle	[95] [96]
PCTK2^S137^, PCTK2^S165^	N/K, N/K	up, up	Potential role in terminally differentiated neurons	[97] [98]
PRPK^T8^	N/K	up	p53/TP53 regulation	[99]

Footnote: Kinases listed here are (^$^) involved in or (^§^) so far not associated with NK cell function and/or CD16 signaling;

1 ^)^UniProt name of kinase and phosphorylation site annotation;

2 ^)^Function of phosphorylation site (A, activation; I, inhibition; brackets indicate possible function based on sequence homology; N/K, not known; *, novel phosphorylation site according to PhosphoSitePlus database);

3 ^)^Direction of phosphorylation site regulation induced by CD16 (up: up-regulation, down: down-regulation, donor-dependent regulation;

4 ^)^Kinase function and.

5 ^)^PubMed references.

Only three of 21 kinases identified as significantly regulated have been previously described as components of the CD16-signaling pathway, i.e. LCK, LYN, and KPCT (PRKCQ/PKCθ). Our data indicate a CD16-induced dephosphorylation of LCK on the inhibitory tyrosine Y192, increased phosphorylation of LCK on the activating region Y394/T395, phosphorylation of LYN on S38, dephosphorylation of LYN on serine S164 and phosphorylation of KPCT on S685. The dephosphorylation of LCK on Y192 and phosphorylation of LYN on S38 were also observed in NK cells co-activated by 2B4 and DNAM-1. Furthermore, kinases implicated in NK cell function, but not specifically assigned to CD16 signaling, i.e. FAK2, FGR, FYN, ITK, and KCC2G/D, were phosphorylated upon CD16 stimulation. FAK2 was dephosphorylated on the amino acids T842 and Y579, which is part of the kinase domain, upon CD16 engagement or 2B4 and DNAM-1 co-engagement. Engagement of CD16 and co-stimulation of 2B4 and DNAM-1 induced phosphorylation of FGR on its supposed auto-phosphorylation site Y412. Triggering of CD16 led to the phosphorylation of FYN on the N-terminal serines S21, S25, and S26, whereas the adjacent Y28 showed a trend toward dephosphorylation (Figure S6). Phosphorylation of these serines was also induced by co-engagement of 2B4 and DNAM-1. Engagement of CD16 as well as co-engagement of 2B4 and DNAM-1 also induced phosphorylation of KCC2G on S381–S384 and KCC2D on S330, in addition to the tentative activation site KCC2G on position 287 (based on sequence homology). For ITK, phosphorylation of Y512 upon CD16 engagement was increased in one donor but decreased in the other one. In contrast, ITK Y512 phosphorylation was reduced following co-engagement of 2B4 and DNAM-1.

The majority of kinases identified by our study have not been characterized in NK cells before. Phosphorylation on the activating Y714 of FER was reduced, as was FES phosphorylation decreased on the activating Y713 by engagement of CD16 or co-engagement of 2B4 and DNAM-1. In contrast, phosphorylation of the proximate S716 residue of FES was significantly induced by engagement of CD16 or co-engagement of 2B4 and DNAM-1. Further, MARK2 phosphorylation was significantly reduced on S456 in NK cells following engagement of CD16 and co-engagement of 2B4 and DNAM-1. Remarkably, PAK4 phosphorylation on S181 was diminished after CD16 engagement, but significantly up-regulated upon co-engagement of 2B4 and DNAM-1. Moreover, NK cell receptor activation led to the phosphorylation of AAK1 and GAK, both of which are involved in clathrin-mediated endocytosis. AAK1 phosphorylation on T620 was significantly enhanced following engagement of CD16 and co-engagement of 2B4 and DNAM-1, whereas the region S672–S678 was reversely regulated. Our data also suggested the phosphorylation of GAK on S829 following CD16 activation. NK cell activation via CD16 and or 2B4 and DNAM-1 co-engagement also led to the phosphorylation of AAPK1 on its predicted auto-phosphorylation site S496, enhanced phosphorylation of GSK3A on the inhibitory site S21, phosphorylation of KPCD2 on S214, phosphorylation of NEK9 on S331-T333 and PCTK2 phosphorylation on S137 and S165. Furthermore, PRPK was phosphorylated on T8 in response to CD16 engagement. In summary, proteome analysis of receptor-induced kinase phosphorylation implicated several new kinases in the regulation of NK cell activation.

## Discussion

The potential of kinase-selective phosphoproteomics (phosphokinomics) to provide comprehensive access to the human kinome has previously been demonstrated [10], [33], [39], [40]. Our results provide a snap-shot of kinase phosphorylation in NK cells shortly after crosslinking of different activating receptors and identify several kinases not previously implicated in NK cells signaling that are regulated downstream of receptor engagement. To our knowledge, this study is the first to employ phosphokinomics to systematically analyze proximal kinase signaling in primary immune cells.

Phosphokinomics is useful for the systematic characterization of kinase-based signaling on a minute time scale [41], [42]. MS-based characterization of phosphorylation events at the level of low abundant protein kinases currently still requires a large amount of cell material and therefore has been restricted to immortalized cell lines. An expansion protocol facilitated the generation of sufficient material (up to 1,5×10^9^ NK cells) for phosphokinome analyses. Importantly, as compared to freshly isolated NK cells, IL-2–cultured NK cells were not significantly altered with respect to the expression of activation receptors, kinetics of intracellular calcium mobilization, or magnitude of degranulation following receptor stimulation. The non-specific kinase inhibitor VI16743 in combination with the commercially available ATP-competitor Purvalanol-B were used to capture the kinome and phosphorylation status of stimulated NK cells. The number of kinases identified by our workflow equaled the number of kinases detected by chemical proteome approaches using a pool of 5 different kinase inhibitors [33], [39], [40], [42] and exceeded the amount of kinases captured by VI16743 alone [33]. MS data verified the expression of 175 protein kinases (and 13 non-protein kinases) from virtually all branches of the human kinome. Although physiological kinase phosphorylation states and activities in primary cells are probably lower than compared to immortalized cell lines, we obtained quantitative information on more than 300 distinct phosphorylation sites. MS-based analysis of phosphopeptide regulation was assisted by a nano-UPLC system enabling the distinct separation of even isobaric phosphopeptides (identical amino acid sequence but different position of modification). This further supported the unambiguous characterization of phosphorylation sites by high-accuracy MS and the exclusion of compromised iTRAQ reporter signals for their quantitative analyses. Finally, statistical evaluation identified kinases significantly regulated by engagement of CD16 or co-engagement of 2B4 and DNAM-1. Our study revealed 21 kinases on which phosphorylation was modulated upon NK cell receptor stimulation. However, not all of the kinase regulations could be confirmed in each experiment. Besides potential variations in human donors, a limited sensitivity and randomized selection of low-abundant peptide ions during LC-MS experiments always cause some variations in the number of identified phosphopeptides, which then obligatory coincide with missing quantitative information.

Many of the regulated kinases presented in this study have not been previously described in NK cells (Table 1 and Figure 6). For most regulated sites, phosphorylation induced by CD16 engagement or 2B4 and DNAM-1 co-engagement demonstrated a similar pattern, in agreement with the notion of a convergence of signals for NK cell activation [26]. However, PAK4 phosphorylation on S181 was induced by co-stimulation of 2B4 and DNAM-1, but reduced following CD16 engagement (Figure 6). Preliminary experiments with single receptor stimulations suggest that PAK4 phosphorylation on S181 is induced by 2B4, but not DNAM-1. Thus, PAK4 may be downstream of 2B4 engagement. In T cells, 2B4 can recruit PIX (PAK interacting exchange factor), a PAK-Rac/Cdc42-specific guanine-exchange factor (GEF), via its adapter protein SAP [43], [44]. Thus, PAK4 might link proximal 2B4 signaling to actin cytoskeleton rearrangements and be dependent on the SAP/PIX adapter complex. In contrast to PAK4, the phosphokinome approach repeatedly revealed consistent regulation of phosphorylation sites on five kinases (FYN, KCC2, AAK1, FES and MARK2) following stimulation with different receptors.

FYN was consistently phosphorylated upon engagement of all receptors investigated on its N-terminal serines S21, S25 and S26, but phosphorylation of the adjacent tyrosine Y28 was unchanged or even reduced. FYN is known to play an important role in proximal signaling by 2B4 [19], [45], [46]. Additionally, FYN is involved in DNAM-1-mediated signaling, as has previously been demonstrated in T cells and mast cells [47], [48]. A role for FYN in NK cell signaling downstream of CD16 has not been described. FYN phosphorylation on S21 and S25 has been identified by other systematic phosphoproteome approaches, whereas FYN phosphorylation on S26 is novel. Interestingly, recent work suggests that FYN is phosphorylated by PKA on S21 and that this phosphorylation can regulate FYN activity and reduce focal adhesion dynamics [49]. Our data support the idea that S21 plays an important functional role: S21 showed a significantly regulated phosphorylation and constituted the most abundant FYN population in comparison to alternative phosphorylations at S25, S26 and Y28. Together, these data point to a central role for FYN in NK cell activation mediated by different activating NK cell receptors.

The calcium/calmodulin-dependent kinase 2 (KCC2, CAMK2) subunits gamma and delta were phosphorylated on S381–S384 and S330 following CD16 engagement or 2B4 and DNAM-1 co-engagement. Furthermore, our data suggests simultaneous phosphorylation of the S287 activation site on KCC2G. Poggi and colleagues have demonstrated that pharmacological inhibition of KCC2 blocks IL-2–cultured NK cell killing of autologous dendritic cells, but not of K562 cells [50]. In T cells, TCR engagement induces translocation of KCC2 to the immune synapse where KCC2 facilitates activation of NFκB through phosphorylation of CARMA1 and Bcl10 [51], [52]. Data also suggest that KCC2 can negatively regulate NFAT activation, diminishing cytokine transcription in T cells [53]. Taken together, although our data implicate activation of KCC2 downstream of activating NK cell receptor engagement, it is not clear how KCC2 modulates NK cell effector functions.

Results revealed that AAK1, the ubiquitously expressed AP2 associated kinase 1 implicated in clathrin-mediated endocytosis [54], [55], possesses a high basal serine/threonine phosphorylation in the region T606-S690. Following engagement of CD16 or co-engagement of 2B4 and DNAM-1, phosphorylation of T672-S678 was reduced. Conversely, stimulation induced AAK1 phosphorylation on T620. Thus, this regulation may potentially serve as molecular switch controlling AAK1 function. AAK1 function has not been studied in NK cells. However, following activation, several NK cell receptors have been shown to undergo SRC-dependent internalization [56]. Moreover, juxtaposed to an exocytic pathway, endocytosis is a prominent feature of the cytotoxic NK cell synapse [57]. The significance and molecular architecture of endocytosis in regard to NK cell cytotoxicity remains to be elucidated.

Stimulation of CD16 or 2B4 and DNAM-1 induced FES phosphorylation of S716 and dephosphorylation of the postulated auto-phosphorylation site Y173 [58], [59], suggesting a regulation of FES activity following NK cell receptor engagement. FES is a SRC family kinase involved in cytoskeleton rearrangements [60], [61]. In mast cells, FES participates in FcεRI-receptor-induced degranulation [62], [63], [64]. Hence, FES might also regulate NK cell degranulation.

MARK2 was consistently dephosphorylated on S456 following engagement of CD16 or co-engagement of 2B4 and DNAM-1. MARK family kinases phosphorylate microtubule-associated proteins, regulate microtubule-based intracellular transport, and are implicated in cellular polarity [65]. In T cells, MARK2 has been shown to become phosphorylated on S400 and T595 following TCR engagement and be required for microtubule-organizing center polarization [66]. Thus, it will be interesting to investigate how MARK2 might contribute to NK cell cytotoxicity.

This study characterizes a significant portion of the NK cell kinome and provides a first unbiased and systematic view into kinase signaling in primary NK cells. Proteomics detected receptor-dependent kinase phosphorylations already after 2 minutes of receptor engagement and allowed the discrimination of differentially phosphorylated kinase populations as demonstrated by FYN. Several kinases were consistently regulated following the engagement of activating receptors. Co-engagement of DNAM-1 and 2B4 generates a phosphorylation pattern at kinases, which was rather similar to that of CD16-stimulated cells, supporting the idea of a core signal network for NK cell activation. Kinases and phosphorylations, which have not previously been implicated in NK cell signaling were regulated and may thus contribute to the process of NK cell activation. Regulation of kinases such as MARK2 and AAK1 are interesting in terms of understanding the molecular pathways for cellular polarization and endocytosis, processes that are induced upon NK cell activation. Thus, contemplating data presented here will direct future studies focused on elucidating the molecular regulation of NK cell effector functions.

## Materials and Methods

### Cells

This study was approved by The Regional Ethics Review Board in Stockholm. Peripheral blood was obtained with informed consent from healthy volunteers. Peripheral blood mononuclear cells (PBMCs) were isolated by density gradient centrifugation (Lymphoprep, Axis-Shield). NK cells were subsequently purified by negative magnetic selection (NK cell isolation kit, Miltenyi Biotec). Freshly isolated NK cells were resuspended in complete medium (RPMI 1640 supplemented with 2 mM L-glutamine and 10% fetal bovine serum [FBS], all Invitrogen) and analyzed within two days after isolation. For polyclonal expansion, purified NK cells (10^5^ per well) and irradiated, allogeneic PBMCs (10^5^ per well) were resuspended in complete medium supplemented with 500 U/ml recombinant IL-2 (Proleukin, Roche) and 10 µg/ml PHA-L (Sigma). Cells were co-cultured in 96-well plates for one week. NK cells were transferred to culture flasks and cultured further for 2 weeks in complete medium supplemented with 50 U/ml IL-2. The purity and viability of NK cells were assessed by flow cytometry prior to analysis. The percentage of CD3^−^CD56^+^ NK cells was greater than 95% in all experiments.

The human erythroleukemia cell line K562 and murine mastocytoma cell line P815 (both American Type Culture Collection) were maintained in complete medium.

### Antibodies and fluorescent reagents

For flow cytometry, NK cell activation, calcium flux analyses, and CD107a degranulation assays, the following mouse monoclonal antibodies (mAbs) were used: anti-CD107a (clone H4A3, IgG1), anti-CD16 (clone 3G8, IgG1), anti-CD56 (clone NCAM16.2, IgG2b, or B159, IgG1), anti-DNAM-1 (clone DX11, IgG1), anti-NKG2D (clone 1D11, IgG1), and anti-CD3 (clone UCHT1, IgG1; all BD Biosciences), anti-2B4 (clone C1.7, IgG1, Beckman Coulter), and isotype control mAb (clone MOPC-21, Sigma-Aldrich). For determination of intracellular calcium mobilization, the calcium-sensitive dyes Fluo-4 and FuraRed (both Molecular Probes) were used.

### Calcium flux analysis

NK cells were resuspended in 50 µl Hanks buffered salt solution (HBSS, Biosource) and incubated with the primary mAbs indicated (1 µg mAb per 10^7^ NK cells) for 30 min on ice. 300 µl staining solution (HBSS supplemented with 1% FBS, 2 µM Fluo-4 AM, 5 µM Fura Red AM, and 5 mM Probenecid) were added, and cells were incubated for an additional 30 minutes at 4°C. Dye-loaded NK cells were washed, resuspended in HBSS, and kept on ice. Prior to analysis NK cells were resuspended in 300 µl HBSS supplemented with 1% FBS, pre-warmed at 37°C in a water bath, and analyzed by flow cytometry. After 30 seconds, samples were removed from the flow cytometer, and F(ab′)_2_-anti-mouse IgG crosslinking antibodies (2 µg Ab per 10^7^ NK cells; Jackson ImmunoResearch) were added. Thereafter, samples were measured for 4 minutes by flow cytometry. Acquired flow cytometric data were analyzed using the FlowJo software (Treestar). NK cells were gated on forward/side scatter, and the ratios of FL-1 and FL-3 intensities were displayed as a function of time.

### CD107a degranulation assay

For assessment of NK cell degranulation, 2×10^5^ K562 cells or 2×10^5^ P815 target cells pre-incubated with 5 µg/ml of the indicated mAbs for 30 minutes at room temperature were mixed with 1×10^5^ NK cells for one hour at 37°C in complete medium. Thereafter, the cells were centrifuged and resuspended in FACS buffer (PBS supplemented with 2% FBS and 2 mM EDTA) containing fluorochrome-conjugated anti-CD56 and anti-CD107a mAbs, and analyzed by flow cytometry. Data were analyzed with FlowJo software.

### IL-2–cultured NK cell stimulation for phosphokinome analysis

For kinome experiments, up to 1,5×10^9^ IL-2-cultured NK cells were used (5×10^8^ NK cells per stimulation). IL-2-cultured NK cells were starved overnight in complete medium without IL-2. Prior to stimulation, cells were resuspended in complete medium at 2×10^7^ cells/ml. NK cells were incubated with primary mAbs directed against CD16, DNAM-1 and 2B4 for 30 minutes on ice (1 µg mAb per 10^7^ NK cells). MAb-coated NK cells were pre-warmed at 37°C in a water bath, thereafter added F(ab′)_2_-anti-mouse-IgG crosslinking antibody (Jackson ImmunoResearch), and stimulated for the indicated time.

### Kinase enrichment from NK cell lysates

Following stimulation, NK cells were lysed in ice-cold lysis buffer containing 50 mM HEPES pH 7.5, 1 M NaCl, 1 mM EGTA, protease inhibitor cocktail (Roche), and 1% Triton-X100. Lysates were centrifuged at 70000× *g* for 30 minutes at 4°C and filtered using a 0.45 µm syringe membrane (Millipore). The protein concentrations of lysates were determined using a Bradford protein assay (BioRad) to ensure equal loading of kinase affinity columns. For small molecule affinity chromatography (SMAC), the kinase inhibitors VI16743 and Purvalanol-B (Tocris) were used. Synthesis of V16743 and generation of both kinase affinity materials were conducted as previously described [10], [41]. Columns (5/50 Tricorn, GE Healthcare) were equilibrated with buffer A (50 mM HEPES-NaOH pH 7.5, 1 M NaCl, 1 mM EGTA, 1 mM EDTA, 0.1% Triton-X100), loaded with NK cell lysates (3 ml/h), washed with 60 column volumes of buffer A (6 ml/h), and equilibrated (2 h, 6 ml/h) with buffer B (50 mM HEPES-NaOH pH 7.5, 1 mM EGTA, 1 mM EDTA, 0.1% Triton-X100). Kinase-enriched fractions were eluted (6 ml/h) separately with 0.5% SDS at room temperature. Eluates were pooled and concentrated by vacuum centrifugation.

### Peptide generation and iTRAQ labeling

Proteins were extracted from concentrated SMAC eluate fractions by chloroform/methanol precipitation [67]. Protein dissolution, denaturation, tryptic digestion, and quantitative peptide labeling by iTRAQ were performed according to the manufacturer's guidelines (Applied Biosystems). Following labeling, peptides derived from IgG isotype control-, CD16 or 2B4 and DNAM-1 co-stimulated NK cells were combined and pooled samples were vacuum-dried, and peptides were desalted using self-packed LiChroprep RP-18 SPE columns (Merck). To quantify kinase phosphorylation induced by CD16, individually, or the co-engagement of 2B4 and DNAM-1, the following iTRAQ-labels were used: experiments I and II (cIgG:114, 2B4/DNAM-1:115, CD16:116), and experiment III (cIgG:114, 2B4/DNAM-1: 117).

### Phosphopeptide enrichment and peptide fractionation

Phosphorylated peptides (phosphopeptides) were enriched from complex peptide samples using the following immobilized metal affinity chromatography (IMAC) protocol: iTRAQ-labeled and desalted peptide mixtures were resolved in IMAC binding buffer (1∶1∶1 methanol∶acetonitrile∶H_2_O containing 2% acetic acid, pH 2.8) and incubated with two Ga^3+^-chelated gel disks (Pierce) for one hour at room temperature or overnight at 4°C. The Ga^3+^-matrix was washed 3 times with 100 µl IMAC-binding buffer to remove unmodified peptides. Retained phosphopeptides were eluted with 600 µl IMAC elution buffer (100 mM ammonium phosphate buffer, pH 4.5). The phosphopeptide-enriched fraction was vacuum-dried, desalted and analyzed by LC-MS/MS. IMAC flow-through and washing fractions were collected and constituted the phosphopeptide-depleted fraction. The phosphopeptide-depleted fraction was further sub-fractioned by SCX chromatography. Thereafter, desalted peptides were resolved and separated on a MonoS PC1.6/5 column (GE Healthcare) using an Ettan micro LC system (GE Healthcare). SCX-separated peptide fractions were vacuum-dried, desalted, and analyzed separately by LC-MS/MS.

### LC-MS/MS analysis and database search

LC-MS/MS analyses of phosphopeptide-enriched and -depleted fractions were performed on an Acquity Ultra Performance LC system (Waters Corp.) connected to an LTQ Orbitrap XL Fourier transform mass spectrometer (Thermo Scientific). Raw MS data were converted to data formats compatible with Mascot search engine (Matrix Science). Data from all peptide fractions were merged using Mascot Daemon (version 2.1.6). The UniProtKB/Swiss-Prot primary sequence database was used for protein identification (release 2011_03, with 525,997 entries; taxonomy *Homo sapiens* with 20,226 entries). In this study, proteins were only considered if they were identified at least with one unique peptide having an individual Mascot peptide score above 20, which indicated identity or extensive homology (p<0.05) using the following Mascot search parameters: enzyme, trypsin (specificity: K/R); maximum missed cleavages, 1; fixed modifications, iTRAQ 4-plex (K), iTRAQ (N terminus), Methylthio (C); variable modifications, phosphorylation (S, T, Y), oxidation (M); peptide tolerance, 10 ppm; MS/MS tolerance, 0.1 Da. Proteins identified by one peptide and Mascot-aided assignment of tyrosine (Y) phosphorylation (n = 21) were checked by manual inspection of corresponding MS/MS spectra, whereas annotation of threonine (T, n = 63) and serine (S, n = 209) phosphorylation was statistically validated by the AScore algorithms [68]. False discovery rates (FDRs) were calculated using the software Scaffold (version Scaffold_3_00_06). On average peptide FDRs less than 1.4% were determined. All MS-data associated with this manuscript are published in the PROteomics IDEntifications Database (PRIDE) (www.ebi.ac.uk/pride/).

### Evaluation of quantitative LC-MS/MS data

Statistical evaluation of quantitative peptide data was conducted by iTRAQassist, a MS device-specific noise algorithm, as previously described [38]. Briefly, statistical evaluation was performed on the basis of spectral data derived from Mascot result files (.dat files) and restricted to peptides uniquely and unambiguously identified within the particular MS/MS data set (peptide tolerance: 10 ppm; MS/MS tolerance: 0.1 Da; iTRAQ reporter mass delta: 0.02 Da). ITRAQassist combines iTRAQ by-product correction and normalization of iTRAQ intensities with a noise-specific algorithm allowing determination of a weighted cumulative regulation factor for each unique peptide and prediction of possible deviations from the calculated regulation values dependent on iTRAQ reporter quality. ITRAQ reporter intensities were re-calculated according to certified and experimentally confirmed ratios of isotopic impurities of the different iTRAQ reporter molecules. The subsequent normalization was accomplished by comparing the trimmed mean of reporter intensities in all samples and discarding the upper and lower 20% of reporter intensities. Peptide regulations were depicted as likelihood curves for every peptide. Peptides containing phosphorylated amino acids were accepted as differentially regulated if their corresponding likelihood curves (phosphopeptide curve) were clearly separated from the cluster of non-phosphorylated peptides belonging to the same protein (protein curve). Regulated and discussed phosphopeptide sequence qualities and phosphorylation site annotations were examined manually.

## Supporting Information

Figure S1 **Purity of IL-2–cultured primary NK cells.** (**A**) PBMC obtained from human peripheral blood by Ficoll gradient centrifugation and (**B**) IL-2–cultured NK cell were stained with fluorochrome-conjugated anti-CD3 and anti-CD56 mAbs and analyzed by flow cytometry. IL-2–cultured NK cell were 95 to 99% CD3^−^CD56^+^ as determined by flow cytometry. Data represent IL-2–cultured NK cell populations used in the phosphoproteome studies.
(PDF)

Figure S2 **VI16743/Purvalanol-B-based affinity chromatography permits comprehensive enrichment of human protein kinases.** (**A**) Kinases were significantly enriched by VI16743/Purvalanol-B-based affinity chromatography. Percentages of kinases and non-kinases (others: proteins without kinase activity) after VI16743/Purvalanol-B-mediated affinity purification are shown. (**B**) Kinases are identified based on a significantly higher number of MS spectra reflecting a better peptide identification and coverage of kinases achieved by MS. Assigned MS spectra per kinase and non-kinases (others) are depicted as mean values ± standard deviation (SD) of all conducted kinase-selective phosphoproteome experiments. Differences between two groups were examined using the Student's t-test (***, p<0.001).
(PDF)

Figure S3 **Statistical validation of phosphorylation site regulation at protein kinases by iTRAQassist.** Statistical evaluation of quantitative peptide data was conducted by iTRAQassist, a MS device specific noise algorithm, as previously described [38]. Phosphorylation sites were accepted as differentially regulated if their corresponding likelihood curves (phosphopeptide curve, green) were clearly separated from the cluster of non-phosphorylated peptides belonging to the same protein (protein curve, gray).
(PDF)

Figure S4 **Number of kinase phosphorylation sites identified in respective experiments.**
(PDF)

Figure S5 **Distribution of iTRAQ-based protein and phosphorylation site regulation factors.** This figure provides a general overview of all protein (black curve) and phosphorylation site (red curve) regulation factors calculated for two representative proteome experiments (I and II, see Table S1). Protein regulations are given as cumulative regulation values calculated on the basis of all non-phosphorylated peptides belonging to the same kinase. The majority of non- and phosphorylated peptides were expectedly not regulated. Protein regulation factors showed a normal distribution, whereas the phosphorylation site curve exhibits a non-normal distribution and was clearly shifted towards positive regulation values.
(PDF)

Figure S6 **ITRAQ-MS-based quantification of N-terminal FYN phosphorylation induced by the engagement of CD16 or 2B4 and DNAM-1.** Distinct phosphopeptide populations were resolved by nano-UPLC (Figure 5) and separately analyzed by mass spectrometry. The figure shows MS/MS fragmentation spectra of distinct FYN phosphopeptides originating from the same peptide sequence (DGSLNQSSGYR). FYN identification and phosphorylation site annotation are based on fragment ions of the b- and y-series (color code: red, b-ion; purple, b-ions minus 98; blue, y-ions; turquoise, y-ions minus 98; green, intensities of iTRAQ reporters or parent ions or b-ions minus H_2_O). Relative iTRAQ intensities of the corresponding phosphopeptides are shown at the right. FYN was differentially phosphorylated at S21, S25, S28 and Y28 following engagement of CD16 or co-engagement of 2B4 and DNAM-1.
(PDF)

Table S1 **Performed phosphokinome experiments.** Footnote: In experiment I and II cIgG-, CD16- and 2B4 and DNAM-1 co-activated NK cells were analyzed. In experiment III cIgG- and 2B4 and DNAM-1 co-activated NK cells were analyzed. ^§^Mascot-based annotation of serine and threonine phosphorylation was checked by the AScore algorithm. 47 phosphorylation sites could not be assigned unambiguously.
(PDF)

Table S2 **Kinases expressed in human NK cells.** Footnote: *Kinase grouping according to Manning et al., 2002.
(PDF)

Table S3 **Kinase phosphorylation in human NK cells.** Footnote: ^1^Serine/threonine annotations by Mascot were validated using AScore algorithm [68]; ^a^predicted phosphorylation site (UniProt); *not listed in UniProt database; when phosphorylation sites could not be assigned unambiguously, alternative sequences are given. Note: Only for peptides containing more than one single serine/threonine residue AScore is required and annotation of tyrosine phosphorylation is not evaluated by AScore algorithm, but was inspected manually.
(PDF)
