# Supplementary figures and images for: Kinome Analysis of Receptor-Induced Phosphorylation in Human Natural Killer Cells
Source: PLoS One. 2012 Jan 4;7(1):e29672. doi: 10.1371/journal.pone.0029672 (PMC3251586; doi:10.1371/journal.pone.0029672)

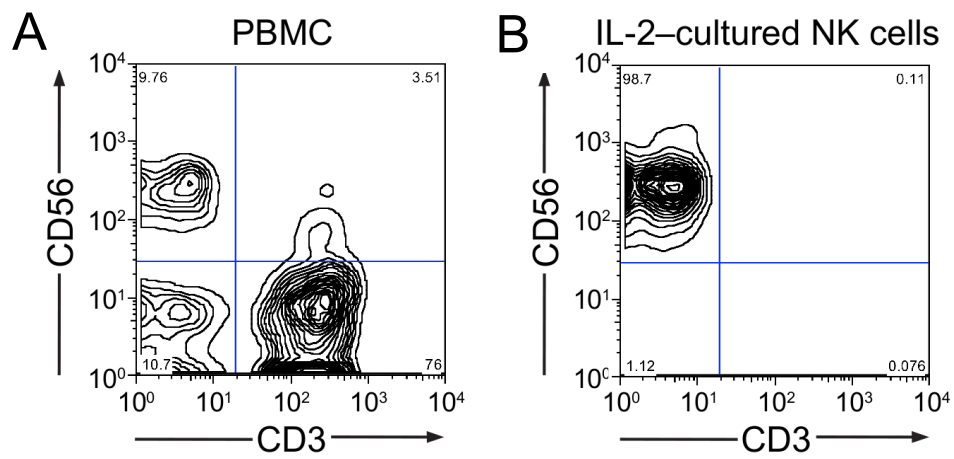

Figure S1, König *et al.*

Supplement: Figure S1 — Purity of IL-2–cultured primary NK cells. (A) PBMC obtained from human peripheral blood by Ficoll gradient centrifugation and (B) IL-2–cultured NK cell were stained with fluorochrome-conjugated anti-CD3 and anti-CD56 mAbs and analyzed by flow cytometry. IL-2–cultured NK cell were 95 to 99% CD3−CD56+ as determined by flow cytometry. Data represent IL-2–cultured NK cell populations used in the phosphoproteome studies. (PDF) [file pone.0029672.s001.pdf]

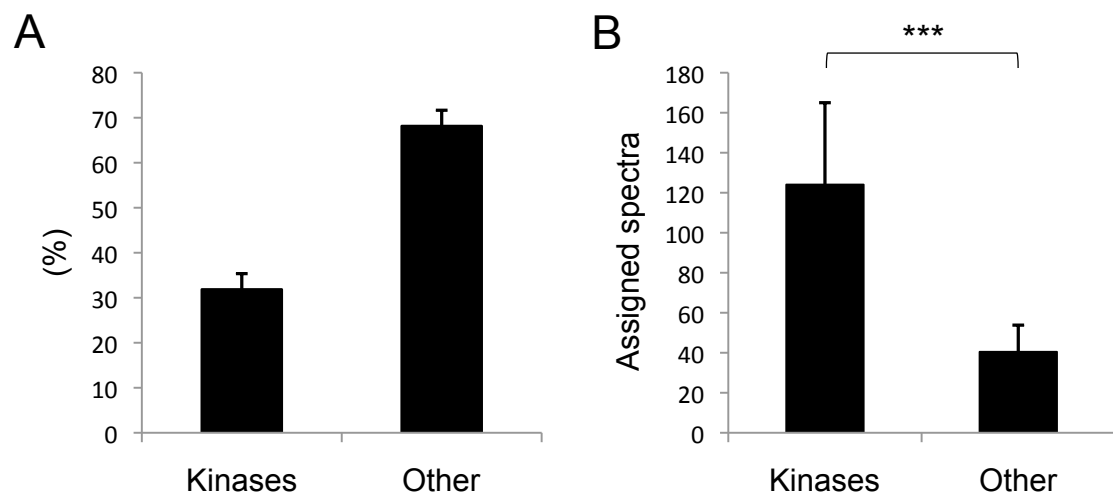

Figure S2, König *et al.*

Supplement: Figure S2 — VI16743/Purvalanol-B-based affinity chromatography permits comprehensive enrichment of human protein kinases. (A) Kinases were significantly enriched by VI16743/Purvalanol-B-based affinity chromatography. Percentages of kinases and non-kinases (others: proteins without kinase activity) after VI16743/Purvalanol-B-mediated affinity purification are shown. (B) Kinases are identified based on a significantly higher number of MS spectra reflecting a better peptide identification and coverage of kinases achieved by MS. Assigned MS spectra per kinase and non-kinases (others) are depicted as mean values ± standard deviation (SD) of all conducted kinase-selective phosphoproteome experiments. Differences between two groups were examined using the Student's t-test (***, p<0.001). (PDF) [file pone.0029672.s002.pdf]

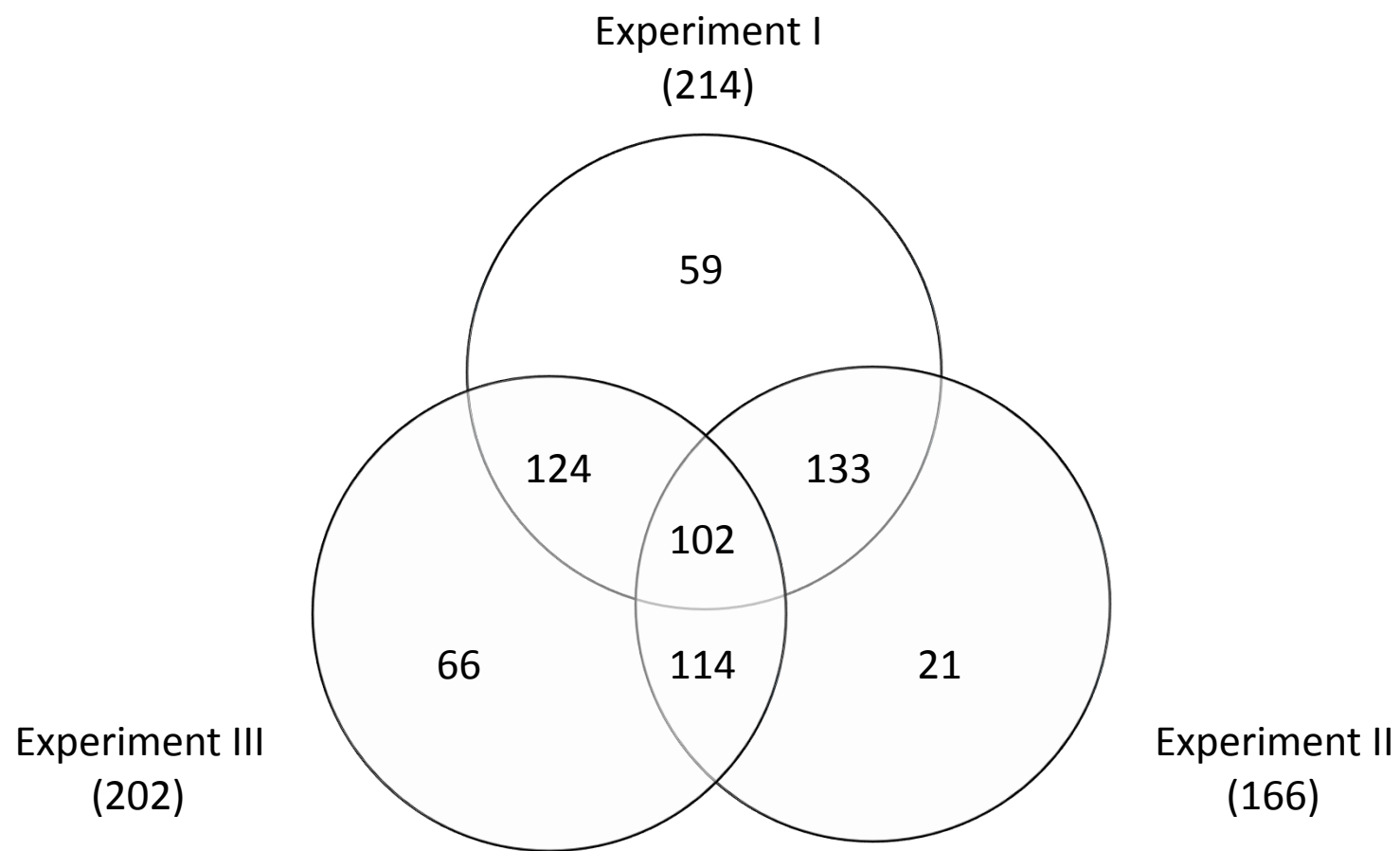

Figure S4, König *et al.*

Supplement: Figure S4 — Number of kinase phosphorylation sites identified in respective experiments. (PDF) [file pone.0029672.s004.pdf]

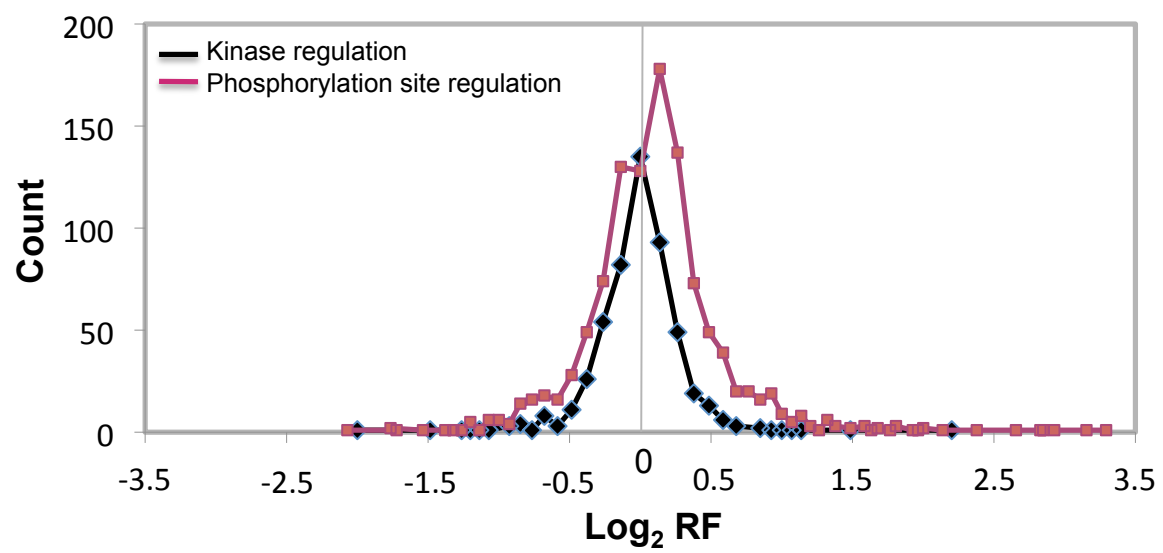

Figure S5, König *et al.*

Supplement: Figure S5 — Distribution of iTRAQ-based protein and phosphorylation site regulation factors. This figure provides a general overview of all protein (black curve) and phosphorylation site (red curve) regulation factors calculated for two representative proteome experiments (I and II, see Table S1). Protein regulations are given as cumulative regulation values calculated on the basis of all non-phosphorylated peptides belonging to the same kinase. The majority of non- and phosphorylated peptides were expectedly not regulated. Protein regulation factors showed a normal distribution, whereas the phosphorylation site curve exhibits a non-normal distribution and was clearly shifted towards positive regulation values. (PDF) [file pone.0029672.s005.pdf]

# FYN\_HUMAN - MS/MS spectra

## iTRAQ reporter

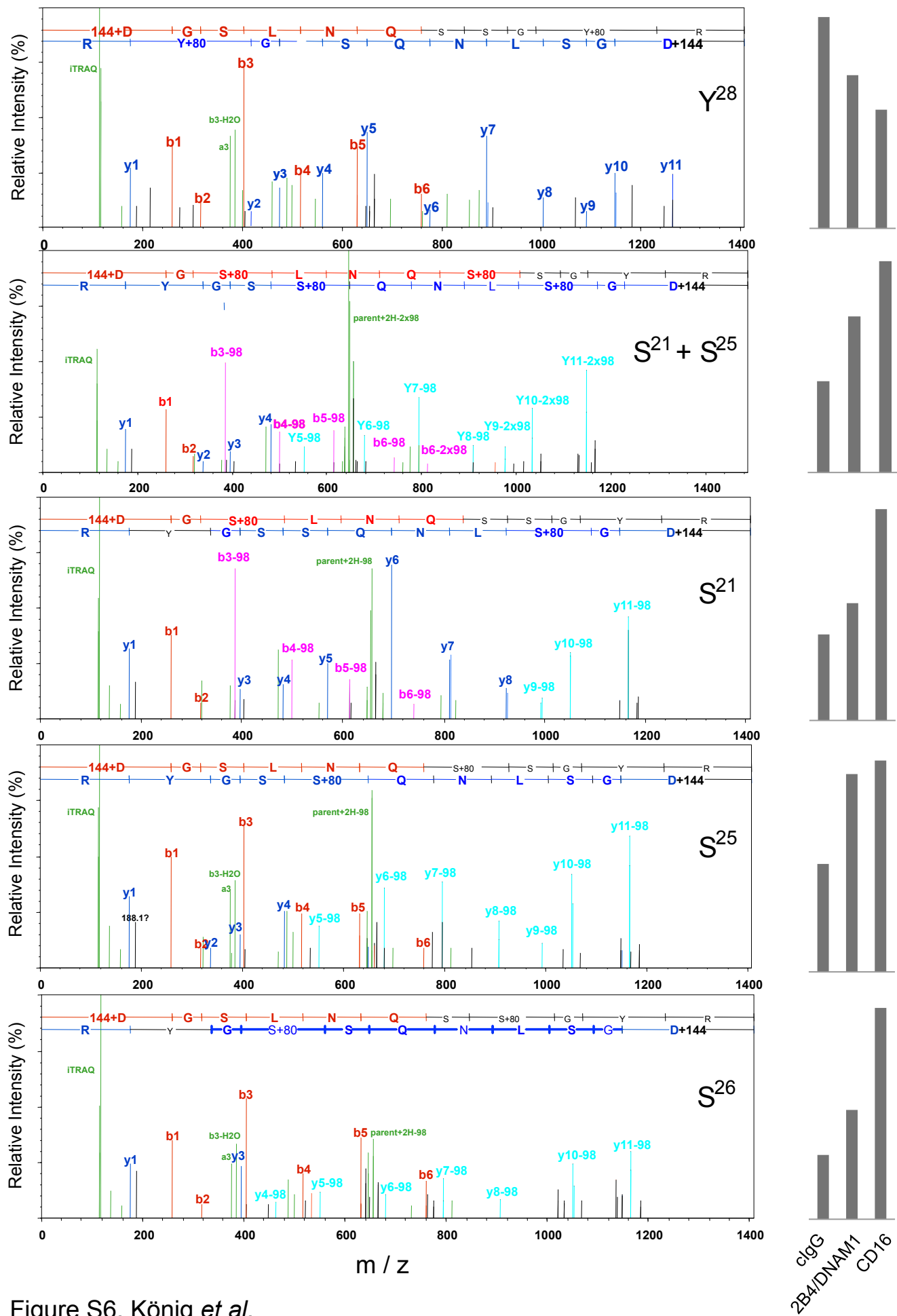

Figure S6, König *et al.*

Supplement: Figure S6 — ITRAQ-MS-based quantification of N-terminal FYN phosphorylation induced by the engagement of CD16 or 2B4 and DNAM-1. Distinct phosphopeptide populations were resolved by nano-UPLC (Figure 5) and separately analyzed by mass spectrometry. The figure shows MS/MS fragmentation spectra of distinct FYN phosphopeptides originating from the same peptide sequence (DGSLNQSSGYR). FYN identification and phosphorylation site annotation are based on fragment ions of the b- and y-series (color code: red, b-ion; purple, b-ions minus 98; blue, y-ions; turquoise, y-ions minus 98; green, intensities of iTRAQ reporters or parent ions or b-ions minus H2O). Relative iTRAQ intensities of the corresponding phosphopeptides are shown at the right. FYN was differentially phosphorylated at S21, S25, S28 and Y28 following engagement of CD16 or co-engagement of 2B4 and DNAM-1. (PDF) [file pone.0029672.s006.pdf]
