# Supplementary material for: Kinome Analysis of Receptor-Induced Phosphorylation in Human Natural Killer Cells
Source: PLoS One. 2012 Jan 4;7(1):e29672. doi: 10.1371/journal.pone.0029672 (PMC3251586; doi:10.1371/journal.pone.0029672)
Supplement: Figure S3 — Statistical validation of phosphorylation site regulation at protein kinases by iTRAQassist. Statistical evaluation of quantitative peptide data was conducted by iTRAQassist, a MS device specific noise algorithm, as previously described [38]. Phosphorylation sites were accepted as differentially regulated if their corresponding likelihood curves (phosphopeptide curve, green) were clearly separated from the cluster of non-phosphorylated peptides belonging to the same protein (protein curve, gray). (PDF) [file pone.0029672.s003.pdf]

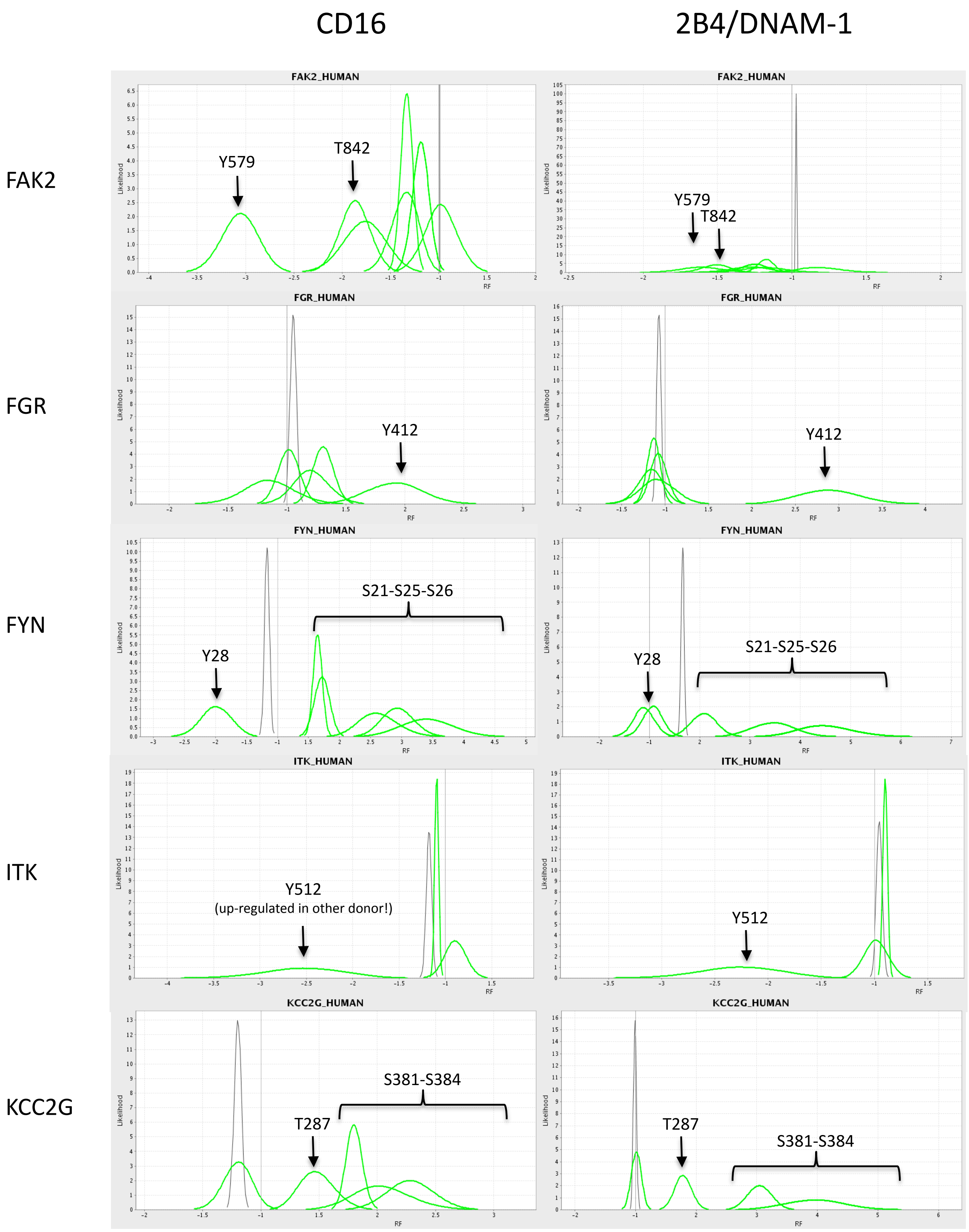

Figure S3, König *et al.*

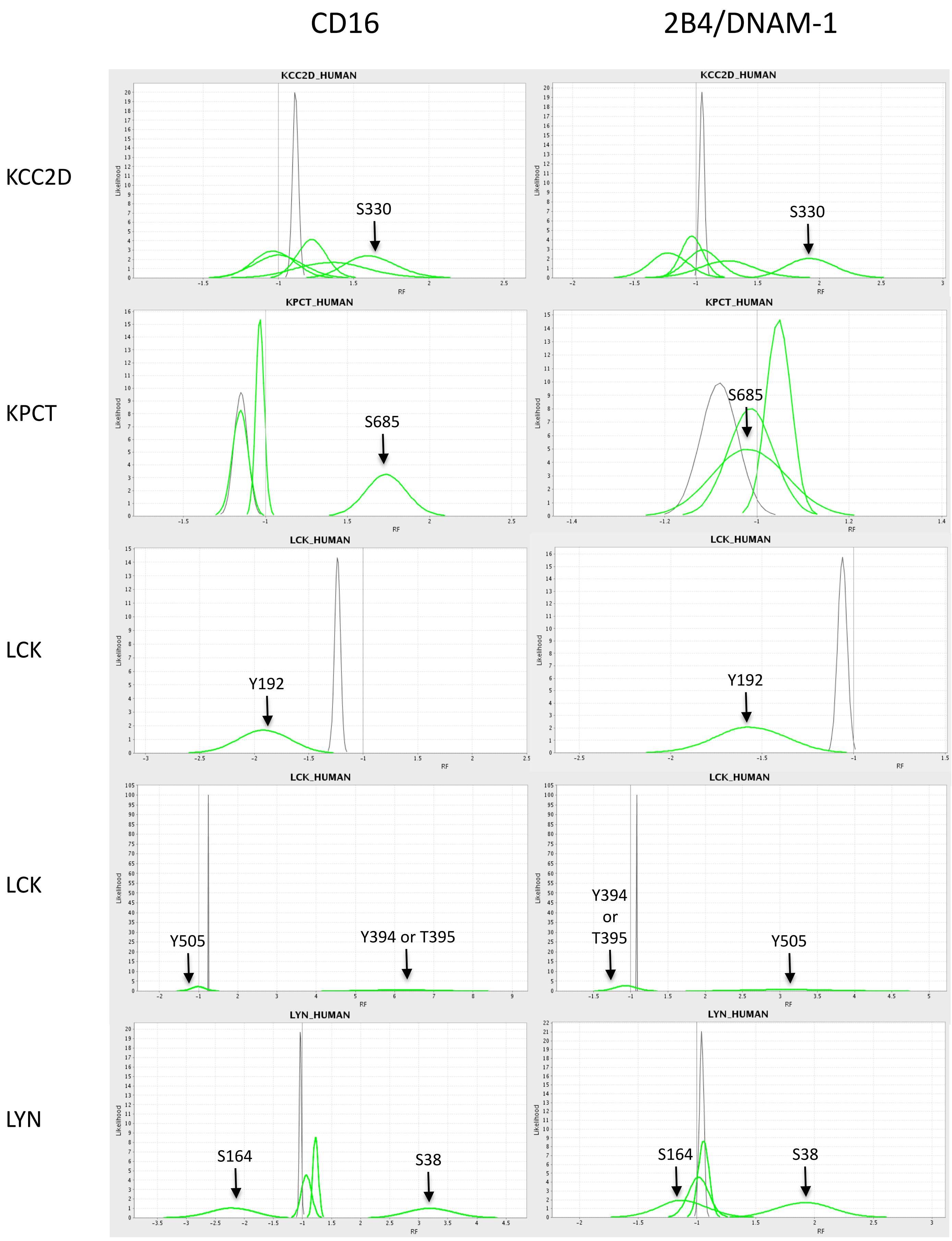

Figure S3, König *et al.*

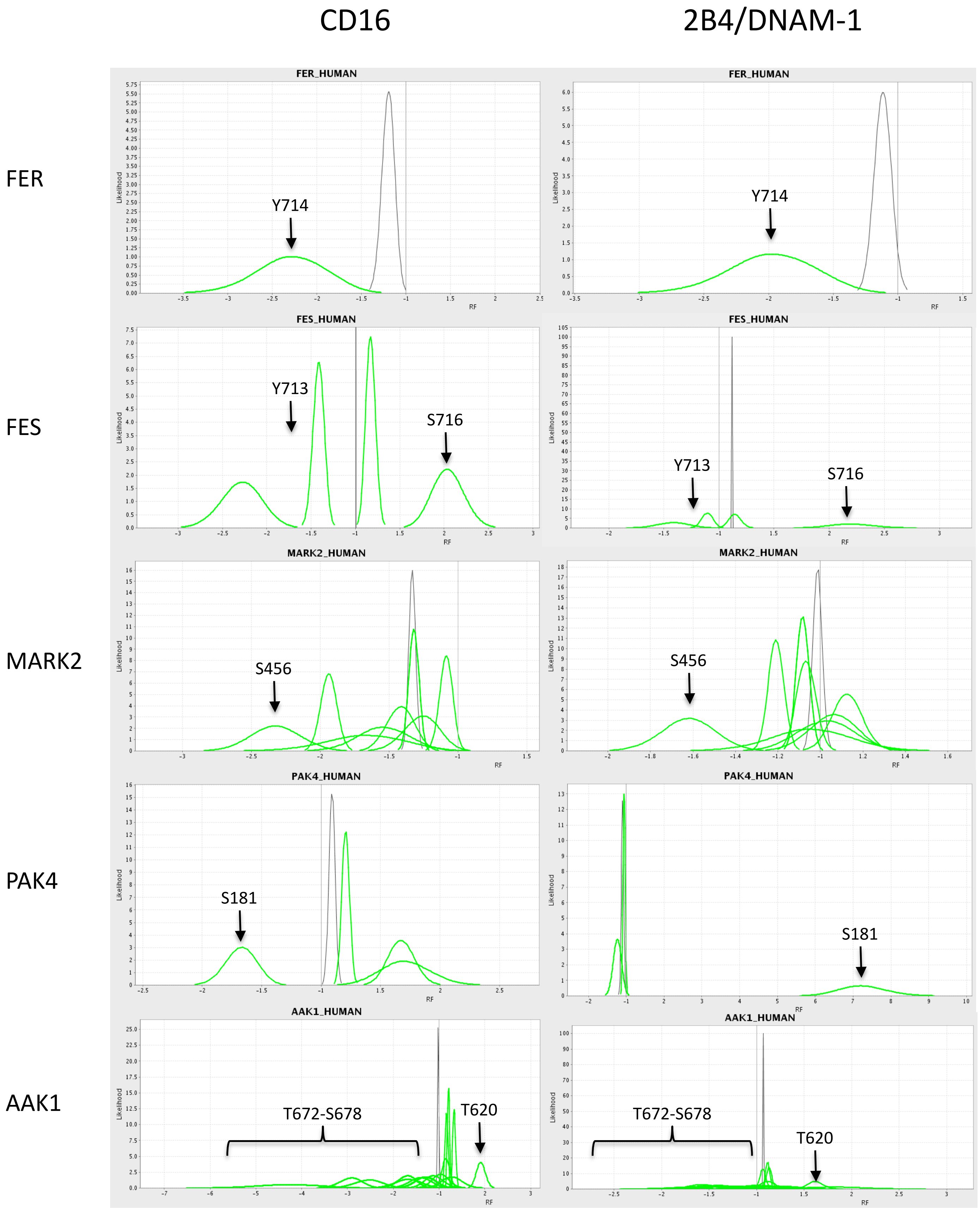

Figure S3, König *et al.*

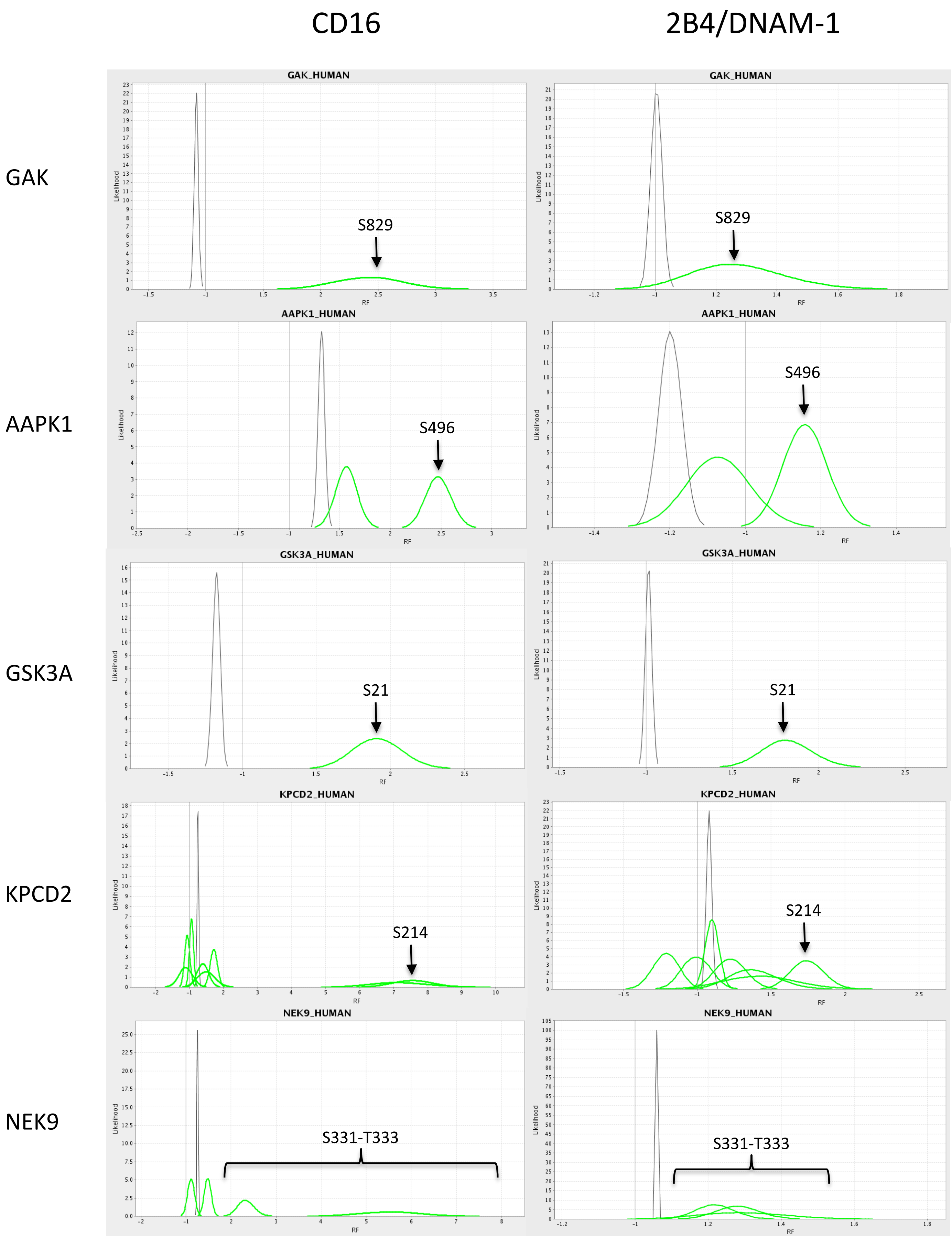

Figure S3, König *et al.*

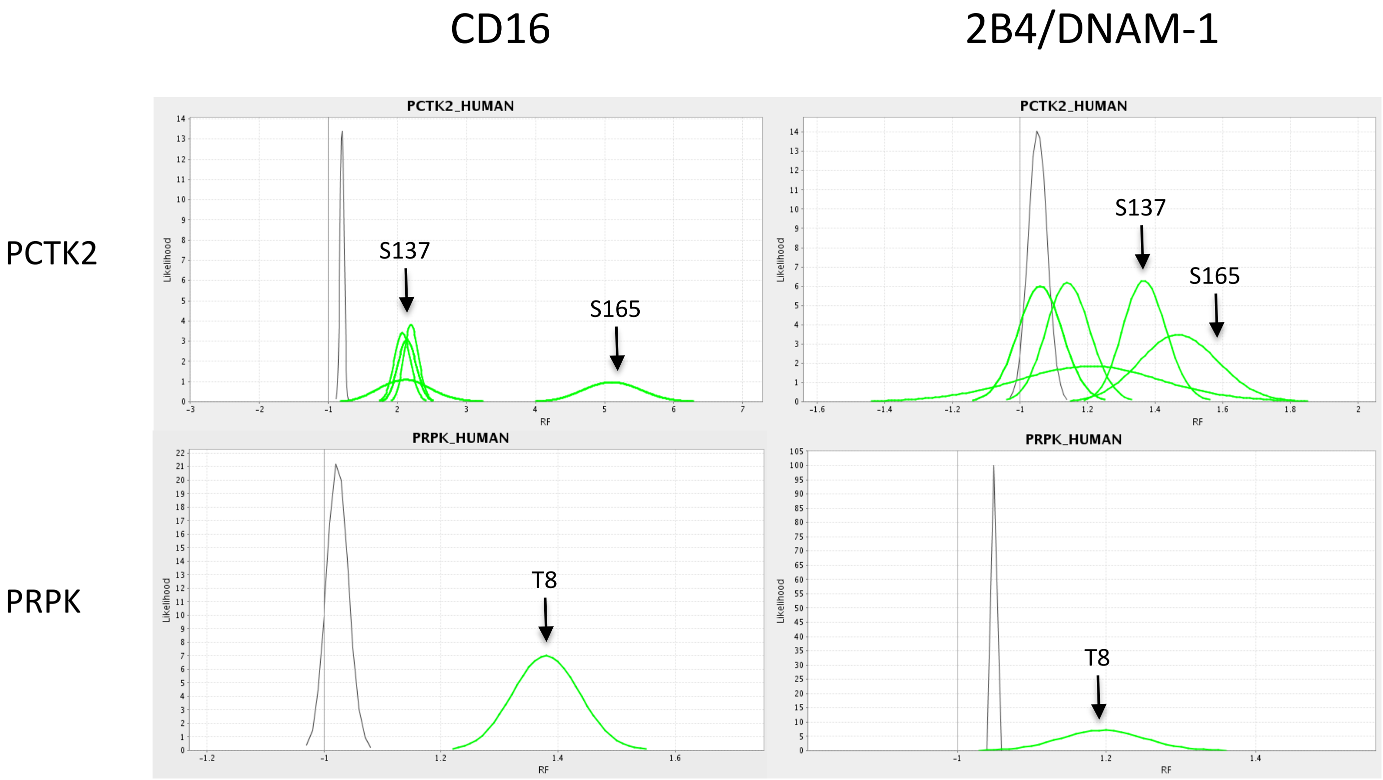

Figure S3, König *et al.*
