# Supplementary material for: Kinome Analysis of Receptor-Induced Phosphorylation in Human Natural Killer Cells
Source: PLoS One. 2012 Jan 4;7(1):e29672. doi: 10.1371/journal.pone.0029672 (PMC3251586; doi:10.1371/journal.pone.0029672)
Supplement: Table S1 — Performed phosphokinome experiments. Footnote: In experiment I and II cIgG-, CD16- and 2B4 and DNAM-1 co-activated NK cells were analyzed. In experiment III cIgG- and 2B4 and DNAM-1 co-activated NK cells were analyzed. §Mascot-based annotation of serine and threonine phosphorylation was checked by the AScore algorithm. 47 phosphorylation sites could not be assigned unambiguously. (PDF) [file pone.0029672.s007.pdf]

Table S1 - Performed phosphokinome experiments

Experiment	Kinases	Phosphorylated kinases	Phosphorylation sites
I (Donor 1)	144	92	214
II (Donor 2)	149	94	166
III (Donor 1)	153	89	202
Total	188	95	313[§]

In experiment I and II cIgG-, CD16- and 2B4/DNAM-1-activated NK cells were analyzed. In experiment III cIgG- and 2B4/DNAM-1-activated NK cells were analyzed. [§]Mascot-based annotation of serine and threonine phosphorylation was checked by the AScore algorithm. 47 phosphorylation sites could not be assigned unambiguously.
