# Supplementary material for: Kinome Analysis of Receptor-Induced Phosphorylation in Human Natural Killer Cells
Source: PLoS One. 2012 Jan 4;7(1):e29672. doi: 10.1371/journal.pone.0029672 (PMC3251586; doi:10.1371/journal.pone.0029672)
Supplement: Table S2 — Kinases expressed in human NK cells. Footnote: *Kinase grouping according to Manning et al., 2002. (PDF) [file pone.0029672.s008.pdf]

Table S2 - Kinases expressed in human NK cells

UniProt name	UniProt accession	Mascot score	Kinase family*	Number of phosphorylation sites	Frequency of identification
AAK1	Q2M2I8	29382,11	Other	16	3
AAKB1	Q9Y478	4170,08	CAMK	1	3
AAKB2	O43741	692,88	CAMK	1	3
AAKG1	P54619	1944,86	CAMK	0	3
AAPK1	Q13131	13246,47	CAMK	6	3
ABL1	P00519	87,56	TK	1	1
ABL2	P42684	498,71	TK	3	3
ACK1	Q07912	183,59	TK	2	3
ACVR1	Q04771	199,49	TKL	0	3
ATM	Q13315	144,81	Atypical	1	2
ATR	Q13535	43,66	Atypical	0	2
AURKB	Q96GD4	3834,9	Other	0	3
BMP2K	Q9NSY1	507,74	Other	1	3
BMPR2	Q13873	25,42	TKL	0	2
BRAF1	P15056	58,59	TKL	0	1
BUB1	O43683	23,06	Other	0	1
CD2L5	Q14004	253,18	CMGC	2	3
CD2L6	Q9BWU1	177,83	CMGC	0	3
CD2L7	Q9NYV4	1407,36	CMGC	5	3
CDC2	P06493	9114,32	CMGC	1	3
CDK10	Q15131	637,15	CMGC	1	3
CDK2	P24941	9604,51	CMGC	0	3
CDK3	Q00526	424,72	CMGC	0	3
CDK4	P11802	55,21	CMGC	0	3
CDK5	Q00535	8252,57	CMGC	1	3
CDK6	Q00534	12642,49	CMGC	0	3
CDK7	P50613	7967,32	CMGC	2	3
CDK8	P49336	304,18	CMGC	0	2
CDK9	P50750	19939,1	CMGC	1	3
CDKL5	O76039	2230,24	CMGC	0	2
CHKA	P35790	66,71	NPK	0	2
CHKB	Q9Y259	130,99	NPK	1	2
CLK1	P49759	3288,81	CMGC	2	3
CLK2	P49760	831,4	CMGC	3	3
CLK3	P49761	480,8	CMGC	2	3
CLK4	Q9HAZ1	108,23	CMGC	0	3
CSK	P41240	4931,72	TK	0	3
CSK21	P68400	19090,38	Other	0	3
CSK22	P19784	18900,49	Other	1	3
CSK2B	P67870	34579,43	Other	0	3
CTRO	O14578	49,48	AGC	0	1
DAPK1	P53355	322,25	CAMK	0	1
DCLK3	Q9C098	21,21	CAMK	0	1
DYR1A	Q13627	368,32	CMGC	1	3
E2AK4	Q9P2K8	20,96	Other	0	1
EPHA2	P29317	559,86	TK	2	2
EPHA3	P29320	20,72	TK	1	1
EPHA4	P54764	93,87	TK	0	1

ERN1	O75460	102,49	Other	0	3
FAK2	Q14289	68824,25	TK	5	3
FER	P16591	735,11	TK	1	3
FES	P07332	38974,8	TK	7	3
FGR	P09769	7049,04	TK	8	3
FRK	P42685	219,64	TK	0	2
FYN	P06241	4487	TK	4	3
GAK	O14976	14257,64	Other	2	3
GRK6	P43250	38,4	AGC	0	1
GSK3A	P49840	7348,23	CMGC	2	3
GSK3B	P49841	14672,04	CMGC	4	3
HIPK1	Q86Z02	1265,72	CMGC	0	3
HIPK4	Q8NE63	23,47	CMGC	1	1
HUNK	P57058	78,76	CAMK	0	2
IGF1R	P08069	21,25	TK	1	1
IKKE	Q14164	3439,76	Other	1	3
ILK	Q13418	929,41	TKL	0	3
IRAK3	Q9Y616	52,1	TKL	1	2
IRAK4	Q9NWZ3	225,56	TKL	0	3
ITK	Q08881	5802,08	TK	4	3
JAK1	P23458	4371,58	TK	0	3
JAK3	P52333	2068,96	TK	0	3
KAPCA	P17612	44,06	AGC	0	1
KAPCB	P22694	22,11	AGC	0	1
KC1A	P48729	12086,14	CK1	2	3
KC1D	P48730	7595,95	CK1	8	3
KC1E	P49674	1411,24	CK1	3	3
KC1G1	Q9HCP0	1167,78	CK1	0	3
KC1G2	P78368	85,38	CK1	0	1
KC1G3	Q9Y6M4	545,04	CK1	3	3
KCC2D	Q13557	36810,67	CAMK	5	3
KCC2G	Q13555	6369,55	CAMK	5	3
KKCC1	Q8N5S9	28,14	Other	1	1
KPB1	P46020	27,56	NPK	0	1
KPB2	P46019	27,56	NPK	0	1
KPCB	P05771	254,32	AGC	0	2
KPCD2	Q9BZL6	8736,21	CAMK	9	3
KPCD3	O94806	896,12	CAMK	5	3
KPCL	P24723	456,78	AGC	2	3
KPCT	Q04759	3748,25	AGC	5	3
KPYM	P14618	654,22	NPK	0	3
KS6A1	Q15418	10969,49	AGC	4	3
KS6A3	P51812	4708,97	AGC	3	3
KS6A4	O75676	21,73	AGC	1	1
KSR1	Q8IVT5	123,41	TKL	0	1
KSYK	P43405	2344,74	TK	3	3
KT3K	Q9HA64	5995,56	NPK	0	3
LCK	P06239	13146,16	TK	7	3
LIMK1	P53667	3151,75	TKL	4	3
LIMK2	P53671	291,48	TKL	0	3
LRRK2	Q5S007	55,32	TKL	0	1

LYN	P07948	14128,76	TK	6	3
M3K1	Q13233	764,74	STE	5	3
M3K11	Q16584	1965,13	TKL	7	3
M3K2	Q9Y2U5	5021,94	STE	4	3
M3K3	Q99759	2902,63	STE	6	3
M3K5	Q99683	1019	STE	1	3
M4K1	Q92918	1501,1	STE	3	3
M4K4	O95819	1430,18	STE	9	3
M4K5	Q9Y4K4	50,92	STE	0	1
MAPK5	Q8IW41	379,76	CAMK	0	2
MARK2	Q7KZI7	10676,37	CAMK	11	3
MARK3	P27448	1946,1	CAMK	9	3
MELK	Q14680	363,21	CAMK	2	3
MK01	P28482	128935,11	CMGC	1	3
MK03	P27361	7729,69	CMGC	1	3
MK08	P45983	3295,45	CMGC	0	3
MK09	P45984	3085,62	CMGC	0	3
MK13	O15264	66,55	CMGC	0	2
MK15	Q8TD08	2253,1	CMGC	0	2
MLKL	Q8NB16	374,43	TKL	0	2
MLTK	Q9NYL2	1579,77	TKL	4	3
MP2K1	Q02750	394,6	STE	0	3
MP2K2	P36507	87,99	STE	1	3
MP2K4	P45985	53,24	STE	0	1
MP2K6	P52564	332,74	STE	0	3
MRCKA	Q5VT25	22,59	AGC	0	2
MYLK	Q15746	364,82	CAMK	0	2
NAGK	Q9UJ70	44,06	NPK	0	1
NEK1	Q96PY6	322,68	Other	1	2
NEK2	P51955	294,46	Other	1	3
NEK4	P51957	68,31	Other	0	1
NEK5	Q6P3R8	63,55	Other	0	1
NEK6	Q9HC98	53,91	Other	0	1
NEK7	Q8TDX7	249,14	Other	0	1
NEK9	Q8TD19	23350,92	Other	5	3
OBSCN	Q5VST9	31,97	CAMK	0	2
P3C2B	O00750	20,52	NPK	0	1
PAK4	O96013	8035,48	STE	5	3
PCTK1	Q00536	3465,94	CMGC	6	3
PCTK2	Q00537	5398,73	CMGC	7	3
PDK4	Q16654	32,79	Atypical	0	1
PDXK	O00764	27491,67	NPK	0	3
PGK1	P00558	30,05	NPK	0	1
PHKG2	P15735	25,66	CAMK	0	1
PI4KA	P42356	37,9	NPK	0	1
PK3CB	P42338	65,65	NPK	0	1
PK3CD	O00329	8455,18	NPK	0	3
PLK1	P53350	358,07	Other	0	3
PLK4	O00444	91,84	Other	1	3
PRKDC	P78527	68,61	Atypical	0	2
PRP4B	Q13523	3202,33	CMGC	9	3

PRPK	Q96S44	13885,11	Other	2	3
QSK	Q9Y2K2	59,58	CAMK	0	1
RIOK2	Q9BVS4	952,99	Atypical	6	3
RIPK2	O43353	452,85	TKL	0	3
ROCK1	Q13464	494,45	AGC	2	3
ROCK2	O75116	219,45	AGC	0	3
ROS	P08922	63,36	TK	0	1
SLK	Q9H2G2	729,37	STE	1	3
SNRK	Q9NRH2	40,41	CAMK	1	1
SPEG	Q15772	44,55	CAMK	0	1
SRC	P12931	1199,46	TK	0	3
SRPK2	P78362	198,05	CMGC	0	2
STK10	O94804	1172,01	STE	2	3
STK11	Q15831	293,16	CAMK	1	2
STK16	O75716	370,49	Other	0	2
STK3	Q13188	258,11	STE	1	1
STK4	Q13043	11264,42	STE	3	3
STK6	O14965	1393,05	Other	0	3
TAOK1	Q7L7X3	967,95	STE	3	2
TAOK2	Q9UL54	380,15	STE	2	2
TAOK3	Q9H2K8	396,22	STE	1	2
TBK1	Q9UHD2	21667,48	Other	0	3
TEC	P42680	282,17	TK	3	2
TESK2	Q96S53	48,97	TKL	0	1
TEX14	Q8IWB6	34,15	Other	0	2
TGFR1	P36897	628,49	TKL	0	3
TGFR2	P37173	288,15	TKL	0	2
TITIN	Q8WZ42	177,03	CAMK	1	3
TLK2	Q86UE8	67,26	Other	0	1
TNIK	Q9UKE5	1583,1	STE	6	3
TNK1	Q13470	23,03	TK	0	1
TOPK	Q96KB5	30,6	Other	0	1
TSSK2	Q96PF2	292,96	CAMK	0	1
TXK	P42681	1716,77	TK	2	3
TYK2	P29597	288,18	TK	1	3
ULK3	Q6PHR2	256,11	Other	0	3
WEE1	P30291	21,23	Other	1	1
YES	P07947	447,61	TK	0	3

*Kinase grouping according to Manning et al., Science 2002.
