# Supplementary material for: Kinome Analysis of Receptor-Induced Phosphorylation in Human Natural Killer Cells
Source: PLoS One. 2012 Jan 4;7(1):e29672. doi: 10.1371/journal.pone.0029672 (PMC3251586; doi:10.1371/journal.pone.0029672)
Supplement: Table S3 — Kinase phosphorylation in human NK cells. Footnote: 1Serine/threonine annotations by Mascot were validated using AScore algorithm [68]; apredicted phosphorylation site (UniProt); *not listed in UniProt database; when phosphorylation sites could not be assigned unambiguously, alternative sequences are given. Note: Only for peptides containing more than one single serine/threonine residue AScore is required and annotation of tyrosine phosphorylation is not evaluated by AScore algorithm, but was inspected manually. (PDF) [file pone.0029672.s009.pdf]

Table S3 - Kinase phosphorylation in human NK cells

| UniProt name | UniProt accession | Mascot peptide score | Phosphory-lation site | Ascore[1] | Missed cleavage sites | Peptide sequence |
|---|---|---|---|---|---|---|
| AAK1 | Q2M2I8 | 108 | S20* | 0 | | EQGGSGLGSGS~SGGGGSTSGLGSGYIGR, EQGGSGLGSGSS~GGGGSTSGLGSGYIGR |
| AAK1 | Q2M2I8 | 104 | S21[a] | 8 | 1 | REQGGSGLGSGSS~GGGGSTSGLGSGYIGR |
| AAK1 | Q2M2I8 | 71 | S618 | 12 | | VGS~LTPPSSPK |
| AAK1 | Q2M2I8 | 29 | S624 | 10 | | VGSLTPPSS~PK |
| AAK1 | Q2M2I8 | 61 | S637 | 0 | 1 | RILS~DVTHSAVFGVPASK, RILSDVT~HSAVFGVPASK |
| AAK1 | Q2M2I8 | 82 | S652[a] | 9 | | S~TQLLQAAAAEASLNK |
| AAK1 | Q2M2I8 | 39 | S676 | 0 | | SATTTPS~GSPR, SATTTPSGS~PR |
| AAK1 | Q2M2I8 | 59 | S678* | 9 | | SATTTPSGS~PR |
| AAK1 | Q2M2I8 | 69 | T389 | 47 | | AGQTQPNPGILPIQPALT~PR |
| AAK1 | Q2M2I8 | 62 | T605* | 11 | | VQT~TPPPAVQGQK |
| AAK1 | Q2M2I8 | 50 | T606 | 13 | | VQTT~PPPAVQGQK |
| AAK1 | Q2M2I8 | 73 | T620 | 26 | | VGSLT~PPSSPK |
| AAK1 | Q2M2I8 | 56 | T640[a] | 3 | | ILSDVT~HSAVFGVPASK |
| AAK1 | Q2M2I8 | 64 | T672* | 0 | | SAT~TTPSGSPR, SATT~TPSGSPR |
| AAK1 | Q2M2I8 | 44 | T673* | 9 | | SATT~TPSGSPR |
| AAK1 | Q2M2I8 | 36 | T674* | 3 | | SATTT~PSGSPR |
| AAKB1 | Q9Y478 | 25 | S24 | 14 | 1 | RDS~SGGTK |
| AAKB2 | O43741 | 71 | S183[a] | 14 | | DLSS~SPPGPYGQEMYAFR |
| AAPK1 | Q13131 | 24 | S486 | 25 | | S~GTATPQR |
| AAPK1 | Q13131 | 41 | S496 | 43 | | SGS~VSNYR |
| AAPK1 | Q13131 | 39 | S506* | 0 | | S~DSDAEAQGK, SDS~DAEAQGK |
| AAPK1 | Q13131 | 62 | S508 | 43 | | SDS~DAEAQGK |
| AAPK1 | Q13131 | 31 | T488* | 6 | | SGT~ATPQR |
| AAPK1 | Q13131 | 35 | T490 | 16 | | SGTAT~PQR |
| ABL1 | P00519 | 23 | S569 | 26 | | GQGESDPLDHEPAVS~PLLPR |
| ABL2 | P42684 | 55 | S618* | 4 | | GAQASS~GSPALPR |
| ABL2 | P42684 | 47 | S631 | 26 | 1 | DKS~PSSLLEDAK |
| ABL2 | P42684 | 78 | S936 | 26 | | VPVLIS~PTLK |
| ACK1 | Q07912 | 27 | S785 | 9 | | EPLS~PQGSR |
| ACK1 | Q07912 | 37 | S810* | 9 | | LSSS~PGK |
| ATM | Q13315 | 48 | S1883* | 0 | | S~TTPANLDSESEHFFR, ST~TPANLDSESEHFFR |
| BMP2K | Q9NSY1 | 24 | S715* | 23 | | TS~PASK |
| CD2L5 | Q14004 | 38 | S325 | 39 | | DDS~PVSHR |
| CD2L5 | Q14004 | 25 | T1246 | | | ILELT~PEPDRPR |
| CD2L7 | Q9NYV4 | 36 | S1082* | 0 | | NS~SPAPPQPAPGK, NSS~PAPPQPAPGK |
| CD2L7 | Q9NYV4 | 64 | S1083 | 8 | | NSS~PAPPQPAPGK |
| CD2L7 | Q9NYV4 | 23 | S215 | 50 | | TVDS~PK |
| CD2L7 | Q9NYV4 | 20 | S423 | 5 | 1 | ESKGS~PVFLPR |
| CD2L7 | Q9NYV4 | 24 | T893 | 22 | 1 | LYNSEESRPYT~NK |
| CDC2 | P06493 | 62 | T161 | 79 | | VYT~HEVVTLWYR |
| CDK10 | Q15131 | 22 | T196 | | | AYGVPVKPMT~PK |
| CDK5 | Q00535 | 61 | T17 | 43 | | IGEGTYGT~VFK |
| CDK7 | P50613 | 28 | S164 | 32 | | SFGS~PNR |

| | | | | | | |
|---|---|---|---|---|---|---|
| CDK7 | P50613 | 53 | T170 | 84 | | AYT~HQVVTR |
| CDK9 | P50750 | 30 | T186 | 47 | 1 | NSQPNRYT~NR |
| CHKB | Q9Y259 | 21 | S40* | 11 | | ASS~LSR |
| CLK1 | P49759 | 49 | S140 | 44 | | S~VEDDEEGHLICQSGDVLSAR |
| CLK1 | P49759 | 20 | S26* | 9 | | SSS~SHK |
| CLK2 | P49760 | 29 | S493 | 14 | | LWDS~SR |
| CLK2 | P49760 | 33 | S50* | | | EDS~YHVR |
| CLK2 | P49760 | 30 | S98 | 30 | | GDAYYDTDYRHS~YEYQR |
| CLK3 | P49761 | 31 | S157 | 61 | 1 | YRS~PEPDPYLSYR |
| CLK3 | P49761 | 41 | S215 | 34 | 1 | RDS~DTYR |
| CSK22 | P19784 | 50 | S18 | | | VYAEVNS~LR |
| DYR1A | Q13627 | 22 | S529* | 40 | 1 | ARS~DPTHQHR |
| EPHA2 | P29317 | 55 | S901 | 11 | | LPSTSGS~EGVPFR |
| EPHA2 | P29317 | 32 | Y772 | | | VLEDDPEATY~TTSGGK |
| EPHA3 | P29320 | 21 | T791* | 0 | 1 | WT~SPEAIAYRK, WTS~PEAIAYRK |
| FAK2 | Q14289 | 73 | S375 | | | NS~LPQIPMLNLEAR |
| FAK2 | Q14289 | 34 | T842 | 109 | | SPLT~PEK |
| FAK2 | Q14289 | 43 | Y579 | | | YIEDEDY~YK |
| FAK2 | Q14289 | 52 | Y580 | | | YIEDEDYY~K |
| FAK2 | Q14289 | 40 | Y849* | | 1 | EVGY~LEFTGPPQKPPR |
| FER | P16591 | 79 | Y714 | | | QEDGGVY~SSSGLK |
| FES | P07332 | 26 | S408* | 0 | | HS~TSSSEQER, HST~SSSEQER |
| FES | P07332 | 26 | S410* | 0 | | HSTS~SSEQER, HST~SSSEQER |
| FES | P07332 | 47 | S411* | 6 | | HSTSS~SEQER |
| FES | P07332 | 83 | S716* | | | EEADGVYAAS~GGLR |
| FES | P07332 | 34 | T421* | 59 | 1 | EGGRT~PTLEILK |
| FES | P07332 | 46 | T423* | 22 | | TPT~LEILK |
| FES | P07332 | 91 | Y713 | | | EEADGVY~AASGGLR |
| FGR | P09769 | 23 | S261* | 26 | | S~SITLER |
| FGR | P09769 | 22 | S262* | 23 | | SS~ITLER |
| FGR | P09769 | 31 | S27* | 101 | | S~YGAADHYGPDPTK |
| FGR | P09769 | 33 | S27*, Y34 | 67 | | S~YGAADHY~GPDPTK |
| FGR | P09769 | 26 | T264* | 25 | | SSIT~LER |
| FGR | P09769 | 32 | Y28 | | | SY~GAADHYGPDPTK |
| FGR | P09769 | 65 | Y34 | | | SYGAADHY~GPDPTK |
| FGR | P09769 | 63 | Y412 | | 1 | LIKDDEY~NPCQGSK |
| FYN | P06241 | 53 | S21 | 81 | | DGS~LNQSSGYR |
| FYN | P06241 | 48 | S25 | 26 | | DGSLNQS~SGYR |
| FYN | P06241 | 47 | S26* | 8 | | DGSLNQSS~GYR |
| FYN | P06241 | 33 | Y28* | | | DGSLNQSSGY~R |
| GAK | O14976 | 56 | S829 | 1 | 1 | ESESALMEDRDESEVS~DEGGSPISSEGQEPR |
| GAK | O14976 | 90 | T776* | 0 | | QPGSTAQYDAGAGSPEAEPT~DSDSPPSSSADASR, QPGSTAQYDAGAGSPEAEPTDS~DSPPSSSADASR |
| GSK3A | P49840 | 66 | S21 | 5 | | TSS~FAEPGGGGGGGGGGPGGSASGPGGTGGGK |
| GSK3A | P49840 | 46 | T19* | 0 | | T~SSFAEPGGGGGGGGGGPGGSASGPGGTGGGK, TS~SFAEPGGGGGGGGGGPGGSASGPGGTGGGK |
| GSK3B | P49841 | 48 | S389[a] | 0 | | IQAAAS~TPTNATAASDANTGDR, IQAAAST~PTNATAASDANTGDR |
| GSK3B | P49841 | 35 | S9 | 14 | 1 | TTS~FAESCKPVQQPSAFGSMK |
| GSK3B | P49841 | 66 | T390 | 4 | | IQAAAST~PTNATAASDANTGDR |

| | | | | | | |
|---|---|---|---|---|---|---|
| GSK3B | P49841 | 29 | T7[a] | 0 | 1 | T~TSFAESCKPVQQPSAFGSMK, TT~SFAESCKPVQQPSAFGSMK |
| HIPK4 | Q8NE63 | 23 | T606* | 0 | | GAT~SFLQHVTGHH, GATS~FLQHVTGHH |
| IGF1R | P08069 | 21 | S1339 | | | AS~FDER |
| IKKE | Q14164 | 63 | S664* | 61 | | GAQAS~PPPIAPYPSPTR |
| IRAK3 | Q9Y616 | 22 | S415* | | | NFS~AK |
| ITK | Q08881 | 85 | S565 | 20 | | SNS~EVVEDISTGFR |
| ITK | Q08881 | 40 | T160* | | 1 | KPLPPT~PEDNR |
| ITK | Q08881 | 63 | T513* | 4 | | FVLDDQYT~SSTGTK |
| ITK | Q08881 | 69 | Y512 | | | FVLDDQY~TSSTGTK |
| KC1A | P48729 | 94 | S311 | 8 | | AAQQAAS~SSGQGQQAQTPTGK |
| KC1A | P48729 | 80 | T321 | 25 | | AAQQAASSSGQGQQAQT~PTGK |
| KC1D | P48730 | 49 | S328 | 16 | | GLPS~TASGR |
| KC1D | P48730 | 48 | S331 | 13 | | GLPSTAS~GR |
| KC1D | P48730 | 86 | S382 | 7 | | GAPVNIS~SSDLTGR |
| KC1D | P48730 | 83 | S383 | 0 | | GAPVNISS~SDLTGR, GAPVNISSS~DLTGR |
| KC1D | P48730 | 71 | S384 | 11 | | GAPVNISSS~DLTGR |
| KC1D | P48730 | 24 | S396 | 23 | | MS~TSQIPGR |
| KC1D | P48730 | 48 | T329* | 7 | | GLPST~ASGR |
| KC1D | P48730 | 31 | T349 | 0 | 1 | GTQEVAPPTPLTPT~SHTANTSPRPVSGMER, GTQEVAPPTPLTPTS~HTANTSPRPVSGMER |
| KC1E | P49674 | 27 | S363 | 8 | | IQPAGNTS~PR |
| KC1E | P49674 | 86 | S389 | 13 | | GAPANVS~SSDLTGR |
| KC1E | P49674 | 23 | T362 | 0 | | IQPAGNT~SPR, IQPAGNTS~PR |
| KC1G3 | Q9Y6M4 | 26 | S344 | 0 | | QLPTPVGAVQQDPALS~SNR, QLPTPVGAVQQDPALSS~NR |
| KC1G3 | Q9Y6M4 | 57 | S345* | 5 | | QLPTPVGAVQQDPALSS~NR |
| KC1G3 | Q9Y6M4 | 22 | S413 | 7 | | S~NAPITAPTEVEVMDETK |
| KCC2D | Q13557 | 56 | S330 | 11 | | ES~TESSNTTIEDEDVK |
| KCC2D | Q13557 | 53 | S333 | 3 | | ESTES~SNTTIEDEDVK |
| KCC2D | Q13557 | 68 | S334* | 11 | | ESTESS~NTTIEDEDVK |
| KCC2D | Q13557 | 39 | T336* | 0 | | ESTESSNT~TIEDEDVK, ESTESSNTT~IEDEDVK |
| KCC2D | Q13557 | 77 | T337 | 9 | | ESTESSNTT~IEDEDVK |
| KCC2G | Q13555 | 68 | S311 | 34 | | GAILTTMLVS~R |
| KCC2G | Q13555 | 76 | S381* | 11 | | GS~TESCNTTTEDEDLK |
| KCC2G | Q13555 | 69 | S384 | 8 | | GSTES~CNTTTEDEDLK |
| KCC2G | Q13555 | 30 | T287 | | | QET~VECLR |
| KCC2G | Q13555 | 75 | T382* | 9 | | GST~ESCNTTTEDEDLK |
| KKCC1 | Q8N5S9 | 28 | S52* | 0 | | AAS~VIPGSTSR, AASVIPGS~TSR |
| KPCD2 | Q9BZL6 | 78 | S197 | 20 | 1 | RLS~STSLASGHSVR |
| KPCD2 | Q9BZL6 | 52 | S200 | 14 | | LSSTS~LASGHSVR |
| KPCD2 | Q9BZL6 | 67 | S214 | 2 | | LGTSES~LPCTAEELSR |
| KPCD2 | Q9BZL6 | 38 | S225* | 23 | | S~TTELLPR |
| KPCD2 | Q9BZL6 | 59 | S374* | 8 | | AQS~SLGYIPLMR |
| KPCD2 | Q9BZL6 | 67 | S375 | 11 | | AQSS~LGYIPLMR |
| KPCD2 | Q9BZL6 | 28 | S395* | 0 | 1 | KS~STTLR, KSS~TTLR |
| KPCD2 | Q9BZL6 | 23 | S396 | 14 | | SS~TTLR |
| KPCD2 | Q9BZL6 | 25 | T211* | 14 | | LGT~SESLPCTAEELSR |
| KPCD3 | O94806 | 61 | S364 | 36 | | GLDDTEEPS~PPEDK |
| KPCD3 | O94806 | 34 | S406* | | | VVQS~IK |

| | | | | | | |
|---|---|---|---|---|---|---|
| KPCD3 | O94806 | 53 | S41 | 15 | | LS~NGSFSAPSLTNSR |
| KPCD3 | O94806 | 32 | S414* | 9 | | S~STMVK |
| KPCD3 | O94806 | 36 | S415* | 26 | | SS~TMVK |
| KPCL | P24723 | 57 | S317* | 8 | | TLAGMGLQPGNIS~PTSK |
| KPCL | P24723 | 52 | S675 | 37 | | NFS~YVSPELQP |
| KPCT | Q04759 | 24 | S676 | | | LS~FADR |
| KPCT | Q04759 | 79 | S685 | | | ALINS~MDQNMFR |
| KPCT | Q04759 | 44 | S695 | | | NFS~FMNPGMER |
| KPCT | Q04759 | 34 | T536* | 0 | | T~NTFCGTPDYIAPEILLGQK, TNT~FCGTPDYIAPEILLGQK |
| KPCT | Q04759 | 52 | T538 | 3 | | TNT~FCGTPDYIAPEILLGQK |
| KS6A1 | Q15418 | 63 | S363 | 67 | | DS~PGIPPSAGAHQLFR |
| KS6A1 | Q15418 | 50 | S380 | 36 | 1 | GFS~FVATGLMEDDGKPR |
| KS6A1 | Q15418 | 23 | S732 | 26 | 1 | KLPS~TTL |
| KS6A1 | Q15418 | 23 | T733* | 15 | 1 | KLPST~TL |
| KS6A3 | P51812 | 69 | S369 | 31 | | DS~PGIPPSANAHQLFR |
| KS6A3 | P51812 | 37 | S415 | 15 | | NS~IQFTDGYEVK |
| KS6A3 | P51812 | 48 | S715 | | | NQS~PVLEPVGR |
| KS6A4 | O75676 | 22 | S29* | | | VS~VENFELLK |
| KSYK | P43405 | 60 | S319[a] | 20 | | QESTVS~FNPYEPELAPWAADK |
| KSYK | P43405 | 47 | Y352 | | | EALPMDTEVYESPY~ADPEEIRPK |
| KSYK | P43405 | 32 | Y526 | | | ADENYY~K |
| LCK | P06239 | 64 | S94* | | | ILEQS~GEWWK |
| LCK | P06239 | 75 | T159* | 14 | | ESEST~AGSFSLSVR |
| LCK | P06239 | 59 | T499* | 16 | | SVLEDFFT~ATEGQYQPQP |
| LCK | P06239 | 32 | T501 | 3 | | SVLEDFFTAT~EGQYQPQP |
| LCK | P06239 | 74 | Y192 | | | NLDNGGFY~ISPR |
| LCK | P06239 | 32 | Y394 or T395* | | 1 | LIEDNEY~T~AREGAK |
| LCK | P06239 | 70 | Y505 | | | SVLEDFFTATEGQY~QPQP |
| LIMK1 | P53667 | 23 | S298 | 25 | | SCS~IDR |
| LIMK1 | P53667 | 23 | S307 | 0 | | SPGAGS~LGSPASQR, SPGAGSLGS~PASQR |
| LIMK1 | P53667 | 78 | S310 | 24 | | SPGAGSLGS~PASQR |
| LIMK2 | P53667 | 26 | S289 | 25 | | SNS~ISK |
| LYN | P07948 | 48 | S11 | 50 | | GKDS~LSDDGVDLK |
| LYN | P07948 | 63 | S13 | 34 | | DSLS~DDGVDLK |
| LYN | P07948 | 31 | S164* | 34 | | GS~FSLSVR |
| LYN | P07948 | 36 | S166* | 25 | | GSFS~LSVR |
| LYN | P07948 | 31 | S38* | 14 | | DPTS~NK |
| LYN | P07948 | 20 | T160* | 15 | | ESET~LK |
| M3K1 | Q13233 | 41 | S1018 | 18 | | IPSAS~PQTQR |
| M3K1 | Q13233 | 84 | S21 | 19 | | ATS~PEAGGGGGALK |
| M3K1 | Q13233 | 25 | S266* | 26 | | SES~PGVR |
| M3K1 | Q13233 | 58 | S923 | 23 | | LSAS~SEDISER |
| M3K1 | Q13233 | 75 | T20* | 0 | | AT~SPEAGGGGGALK, ATS~PEAGGGGGALK |
| M3K11 | Q16584 | 60 | S507 | 48 | | ITVQAS~PGLDR |
| M3K11 | Q16584 | 69 | S524 | 8 | | NVFEVGPGDS~PTFPR |
| M3K11 | Q16584 | 38 | S705 | 0 | | TPDS~PPTPAPLLLDLGIPVGQR, PDSPPT~PAPLLLDLGIPVGQR |
| M3K11 | Q16584 | 55 | S740 | 43 | | GGTVS~PPPGTSR |

| | | | | | | |
|---|---|---|---|---|---|---|
| M3K11 | Q16584 | 41 | S748 | 63 | | S~APGTPGTPR |
| M3K11 | Q16584 | 33 | T637* | 25 | | GSSSGT~PK |
| M3K11 | Q16584 | 55 | T702* | 0 | | T~PDSPPTPAPLLLDLGIPVGQR, TPDS~PPTPAPLLLDLGIPVGQR |
| M3K2 | Q9Y2U5 | 39 | S153 | 48 | | LS~IIGPTSR |
| M3K2 | Q9Y2U5 | 53 | S163 | 0 | | S~SPPPGYIPDELHQVAR, SS~PPPGYIPDELHQVAR |
| M3K2 | Q9Y2U5 | 63 | S239 | 25 | | AQS~YPDNHQEFSDYDNPIFEK |
| M3K2 | Q9Y2U5 | 46 | S331 | 29 | | GS~DIDNPTLTVMDISPPSR |
| M3K3 | Q99759 | 39 | S166 | 29 | | HLS~VSSQNPGR |
| M3K3 | Q99759 | 28 | S175* | 0 | | S~SPPPGYVPER, SS~PPPGYVPER |
| M3K3 | Q99759 | 47 | S176* | 17 | | SS~PPPGYVPER |
| M3K3 | Q99759 | 33 | S237 | 14 | | SADS~PSFR |
| M3K3 | Q99759 | 39 | S250 | | | AQS~FPDNR |
| M3K3 | Q99759 | 63 | S340 | 41 | | SADS~ENALSVQER |
| M3K5 | Q99683 | 37 | S958[a] | 9 | | LSALSAGS~NEYLR |
| M4K1 | Q92918 | 38 | S325* | 0 | | SS~SLGIPDADCCR, SSS~LGIPDADCCR |
| M4K1 | Q99683 | 65 | S405* | 0 | | S~PSDEGPGSMGDDGQLSPGVLVR, SPS~DEGPGSMGDDGQLSPGVLVR |
| M4K1 | Q92918 | 35 | S421 | 14 | | SPSDEGPGSMGDDGQLS~PGVLVR |
| M4K4 | O95819 | 35 | S579* | 0 | | TNHS~SPEAQSK, TNHSS~PEAQSK |
| M4K4 | O95819 | 37 | S580* | 9 | | TNHSS~PEAQSK |
| M4K4 | O95819 | 24 | S631 | 40 | | S~PVLSR |
| M4K4 | O95819 | 100 | S639 | 63 | 1 | RDS~PLQGSGQQNSQAGQR |
| M4K4 | O95819 | 36 | S656 | 23 | | NS~TSIEPR |
| M4K4 | O95819 | 30 | S717* | 6 | | SEGSPS~QR |
| M4K4 | O95819 | 55 | S842 | 26 | | QTQS~ASSTLQK |
| M4K4 | O95819 | 23 | T628* | 19 | 1 | TT~SRSPVLSR |
| M4K4 | O95819 | 22 | T840* | 0 | | QT~QSASSTLQK, QTQS~ASSTLQK |
| MARK2 | Q7KZI7 | 25 | S40 | 22 | | GRNS~ATSADEQPHIGNYR |
| MARK2 | Q7KZI7 | 32 | S400* | 43 | | SVS~ANPK |
| MARK2 | Q7KZI7 | 22 | S448* | 0 | 1 | KAS~STAK, KASS~TAK |
| MARK2 | Q7KZI7 | 41 | S456 | | | VPAS~PLPGLER |
| MARK2 | Q7KZI7 | 33 | S486 | 21 | 1 | SRNS~PLLER |
| MARK2 | Q7KZI7 | 45 | S569 | 26 | | VPVAS~PSAHNISSSGGAPDR |
| MARK2 | Q7KZI7 | 26 | S571 | 11 | | VPVASPS~AHNISSSGGAPDR |
| MARK2 | Q7KZI7 | 65 | S619 | 20 | | DQQNLPYGVTPAS~PSGHSQGR |
| MARK2 | Q7KZI7 | 36 | S621 | 0 | | DQQNLPYGVTPASPS~GHSQGR, DQQNLPYGVTPASPSGHS~QGR |
| MARK2 | Q7KZI7 | 51 | S631 | 43 | | GAS~GSIFSK |
| MARK2 | Q7KZI7 | 76 | T466* | 0 | 1 | KT~TPTPSTNSVLSTSTNR, KTT~PTPSTNSVLSTSTNR |
| MARK3 | P27448 | 38 | S397* | 0 | | SSELDAS~DSSSSSNLSLAK, SSELDASDS~SSSSNLSLAK |
| MARK3 | P27448 | 74 | S399* | 0 | | SSELDASDS~SSSSNLSLAK, SSELDASDSS~SSSNLSLAK |
| MARK3 | P27448 | 27 | S431* | 0 | | S~VSSSQK, SVS~SSQK |
| MARK3 | P27448 | 26 | S433* | 19 | | SVS~SSQK |
| MARK3 | P27448 | 29 | S435* | 6 | | SVSSS~QK |
| MARK3 | P27448 | 56 | S481* | 20 | | SSGS~AVGGK |
| MARK3 | P27448 | 53 | S492 | 66 | | GIAPAS~PMLGNASNPNK |
| MARK3 | P27448 | 49 | S563 | 5 | | TPVAS~THSISSAATPDR |
| MARK3 | P27448 | 39 | S624 | 26 | | GS~TNLFSK |

| | | | | | | |
|---|---|---|---|---|---|---|
| MELK | Q14680 | 36 | S505 | 98 | | S~VELDLNQAHMEETPK |
| MELK | Q14680 | 22 | S529 | | | VFGS~LER |
| MK01 | P28482 | 37 | T185, Y187 | 30 | | VADPDHDHTGFLT~EY~VATR |
| MK03 | P27361 | 42 | T202, Y204 | 19 | | IADPEHDHTGFLT~EY~VATR |
| MLTK | Q9NYL2 | 50 | S633 | 36 | | YQQITPVNQS~R |
| MLTK | Q9NYL2 | 36 | S635[a] | 0 | | S~SSPTQYGLTK, SS~SPTQYGLTK |
| MLTK | Q9NYL2 | 72 | S637 | 17 | | SSS~PTQYGLTK |
| MLTK | Q9NYL2 | 48 | S727 | 75 | | VSQSALNPHQS~PDFK |
| MP2K2 | P36507 | 36 | T394 | 26 | | LNQPGT~PTR |
| NEK1 | Q96PY6 | 38 | S1052 | 34 | | TCS~LPDLSK |
| NEK2 | P51955 | 24 | S192* | | | MS~YNEK |
| NEK9 | Q8TD19 | 91 | S331 | 11 | | S~STVTEAPIAVVTSR |
| NEK9 | Q8TD19 | 84 | S332 | 5 | | SS~TVTEAPIAVVTSR |
| NEK9 | Q8TD19 | 26 | S868 | 0 | 1 | VASEAPLEHKPQVEAS~SPR, VASEAPLEHKPQVEASS~PR |
| NEK9 | Q8TD19 | 56 | T254 | 19 | | TFDAT~NPLNLCVK |
| NEK9 | Q8TD19 | 70 | T333 | 8 | | SST~VTEAPIAVVTSR |
| PAK4 | O96013 | 42 | S104 | | | RDS~PPPPAR |
| PAK4 | O96013 | 85 | S148 | 42 | | FAGHSEAGGGS~GDR |
| PAK4 | O96013 | 52 | S167 | 73 | | EGS~GGPQESSR |
| PAK4 | O96013 | 59 | S181 | 8 | 1 | RPLS~GPDVGTPQPAGLASGAK |
| PAK4 | O96013 | 59 | S474 | 30 | | S~LVGTPYWMAPELISR |
| PCTK1 | Q00536 | 40 | S110 | 26 | | KIS~TEDINK |
| PCTK1 | Q00536 | 36 | S12 | 29 | | QLS~MTLR |
| PCTK1 | Q00536 | 28 | S138 | 30 | | LTLNS~PIFDKPLSR |
| PCTK1 | Q00536 | 60 | S153 | 50 | 1 | RVS~LSEIGFGK |
| PCTK1 | Q00536 | 31 | S64* | 0 | | GPLS~SAPEIVHEDLK, GPLSS~APEIVHEDLK |
| PCTK1 | Q00536 | 75 | S95 | 26 | | MGSDGESDQASATSSDEVQS~PVR |
| PCTK2 | Q00537 | 94 | S122 | 11 | | MGSDGESDQASGTSSDEVQS~PTGVCLR |
| PCTK2 | Q00537 | 34 | S137 | | 1 | RIS~MEDLNK |
| PCTK2 | Q00537 | 80 | S165 | 61 | | LQINS~PPFDQPMSR |
| PCTK2 | Q00537 | 41 | S180 | 50 | 1 | RAS~LSEIGFGK |
| PCTK2 | Q00537 | 35 | S506* | 0 | | NS~SYPETGHGK, NSS~YPETGHGK |
| PCTK2 | Q00537 | 23 | S520 | | 1 | RQS~MLF |
| PCTK2 | Q00537 | 29 | S9 | 20 | 1 | RLS~LTLR |
| PLK4 | O00444 | 23 | S665* | 26 | 1 | GFPLADRPPS~PTDNISR |
| PRP4B | Q13523 | 32 | S232* | 36 | 1 | SKS~QDQAR |
| PRP4B | Q13523 | 39 | S257 | 43 | | S~PTDDK |
| PRP4B | Q13523 | 22 | S294 | 26 | 1 | SRS~PVDLR |
| PRP4B | Q13523 | 20 | S368 | 17 | 1 | SRS~PLLNDR |
| PRP4B | Q13523 | 32 | S431 | 41 | 1 | SKDAS~PINR |
| PRP4B | Q13523 | 34 | S580 | 26 | | SPS~PDDILER |
| PRP4B | Q13523 | 31 | S852[a] | 18 | | LCDFGSASHVADNDITPYLVS~R |
| PRP4B | Q13523 | 27 | S93 | | | EGMS~PAK |
| PRP4B | Q13523 | 54 | Y849 | | | LCDFGSASHVADNDITPY~LVSR |
| PRPK | Q96S44 | 95 | T7* | 5 | | AT~TPADGEEPAPEAEALAAAR |
| PRPK | Q96S44 | 38 | T8* | 9 | | ATT~PADGEEPAPEAEALAAAR |
| RIOK2 | Q9BVS4 | 37 | S335 | 8 | | EGSEFS~FSDGEVAEK |
| RIOK2 | Q9BVS4 | 63 | S337 | 20 | | EGSEFSFS~DGEVAEK |

| | | | | | | |
|---|---|---|---|---|---|---|
| RIOK2 | Q9BVS4 | 58 | S350 | 57 | | AEVYGS~ENESER |
| RIOK2 | Q9BVS4 | 34 | S380 | 17 | | SSGDPEQIKEDS~LSEESADAR |
| RIPK2 | Q9BVS4 | 27 | S529 | 0 | | S~PSLNLLQNK, SPS~LNLLQNK |
| RIPK2 | Q9BVS4 | 76 | S531 | 25 | | SPS~LNLLQNK |
| ROCK1 | Q13464 | 21 | S1341* | 19 | | STANQS~FR |
| ROCK1 | Q13464 | 21 | T632* | 0 | | IT~SLQEEVK, ITS~LQEEVK |
| SLK | Q9H2G2 | 65 | S565* | 41 | | VDEDS~AEDTQSNDGK |
| SNRK | Q9NRH2 | 40 | S351* | 26 | | SAS~PSNIK |
| STK10 | O94804 | 76 | S438 | | | QVAEQGGDLS~PAANR |
| STK10 | O94804 | 23 | T185 | | 1 | NLKT~LQK |
| STK11 | Q15831 | 67 | S31[a] | 14 | | IDS~TEVIYQPR |
| STK3 | Q13188 | 75 | S316 | 29 | | ELEEEEENS~DEDELDSHTMVK |
| STK4 | Q13043 | 122 | S320 | 52 | | EVDQDDEENS~EEDEMDSGTMVR |
| STK4 | Q13043 | 36 | S414* | | | S~VPGPLK |
| STK4 | Q13043 | 22 | Y433 | | | IPQDGDY~EFLK |
| TAOK1 | Q7L7X3 | 62 | S421 | 49 | | ASDPQS~PPQVSR |
| TAOK1 | Q7L7X3 | 33 | S445 | 26 | | TAS~LVTR |
| TAOK1 | Q7L7X3 | 30 | S965 | | | NS~PQALR |
| TAOK2 | Q9UL54 | 31 | S473* | 26 | | TAS~LVSR |
| TAOK2 | Q9UL54 | 36 | S486 | | | QIQEHEQDS~ALR |
| TAOK3 | Q9H2K8 | 33 | S442 | 25 | | SAS~LVTR |
| TEC | P42680 | 21 | S308* | 11 | | ETTTS~PK |
| TEC | P42680 | 21 | T306* | 0 | | ETT~TSPK, ETTT~SPK |
| TEC | P42680 | 77 | Y519 | | | YVLDDQY~TSSSGAK |
| TITIN | Q8WZ42 | 21 | T23320* | 7 | | T~PIPQTK |
| TNIK | Q9UKE5 | 33 | S548* | 11 | | QSS~PAMPHK |
| TNIK | Q9UKE5 | 39 | S640 | 17 | | QNS~DPTSENPPLPTR |
| TNIK | Q9UKE5 | 25 | S678 | 0 | | TTS~ISPALAR, TTSIS~PALAR |
| TNIK | Q9UKE5 | 51 | S680 | 11 | | TTSIS~PALAR |
| TNIK | Q9UKE5 | 30 | S707 | | | AS~NPDLR |
| TNIK | P42681 | 25 | S769 | 6 | | SEGS~PVLPHEPAK |
| TXK | P42681 | 91 | Y420 | | | YVLDDEY~VSSFGAK |
| TXK | P42681 | 36 | Y91[a] | | | ALY~DFLPR |
| TYK2 | P29597 | 59 | Y292 | | | LLAQAEGEPCY~IR |
| WEE1 | P30291 | 21 | S642 | 12 | | SVS~LTIY |

[1]Serine/threonine annotations by Mascot were validated using AScore algorithm [76]; [a]predicted phosphorylation site (UniProt); *not listed in UniProt database; when phosphorylation sites could not be assigned unambiguously, alternative sequences are given. Note: Only for peptides containing more than one single serine/threonine residue AScore is required and annotation of tyrosine phosphorylation is not evaluated by AScore algorithm. Mascot search algorithm only considers peptides with two tryptic termini (NTT=2). Peptide delta masses were always less than 0.025 Da.
